# Childhood traumatization is associated with differences in TRPA1 promoter methylation in female patients with multisomatoform disorder with pain as the leading bodily symptom

**DOI:** 10.1186/s13148-019-0731-0

**Published:** 2019-08-28

**Authors:** Johannes Achenbach, Mathias Rhein, Sara Gombert, Fiona Meyer-Bockenkamp, Miro Buhck, Mirjam Eberhardt, Andreas Leffler, Helge Frieling, Matthias Karst

**Affiliations:** 1 Department of Anesthesiology and Intensive Care Medicine, Pain Clinic, Hannover Medical School, Carl-Neuberg-Str. 1, 30625 Hannover, Germany; 2 Laboratory for Molecular Neuroscience, Department of Psychiatry, Social Psychiatry and Psychotherapy, Hannover Medical School, Hannover, Germany

**Keywords:** TRPA1, Methylation, Multisomatoform disorder, Fibromyalgia, Pain, Childhood trauma

## Abstract

**Background:**

The construct of multisomatoform disorder (MSD) is a common point of reference for patients in different somatic and psychosomatic specialties and therefore useful in studying large well-characterized cohorts of a prototype of a somatoform disorder and in parallel as a functional somatic syndrome (FSS). This disorder is characterized by distressing and functionally disabling somatic symptoms with chronic pain as the most frequent and clinically relevant complaint. Pain is perceived by nociceptive nerve fibers and transferred through the generation of action potentials by different receptor molecules known to determine pain sensitivity in pathophysiological processes. Previous studies have shown that for the transient receptor potential ankyrin 1 (TRPA1), receptor methylation of a particular CpG dinucleotide in the promoter region is inversely associated with both heat pain and pressure pain thresholds. In this study, we hypothesized that TRPA1 promoter methylation regulates pain sensitivity of patients with multisomatoform disorder (MSD). A cohort of 151 patients with MSD and 149 matched healthy volunteers were evaluated using quantitative sensory testing, clinical and psychometric assessment, and methylation analysis using DNA isolated from whole blood.

**Results:**

We found CpG -628 to be correlated with mechanical pain threshold and CpG -411 to be correlated with mechanical pain threshold in female volunteers, i.e., higher methylation levels lead to higher pain thresholds. A novel finding is that methylation levels were significantly different between patients with no and severe levels of childhood trauma. CpG methylation also correlated with psychometric assessment of pain and pain levels rated on a visual analog scale.

**Conclusion:**

Our findings support the hypothesis that epigenetic regulation of TRPA1 plays a role in mechanical pain sensitivities in healthy volunteers. They further provide evidence for the possible influence of childhood traumatic experiences on the epigenetic regulation of TRPA1 in patients with MSD.

**Electronic supplementary material:**

The online version of this article (10.1186/s13148-019-0731-0) contains supplementary material, which is available to authorized users.

## Background

If investigation of a patient’s painful symptoms does not reveal a satisfactory somatic diagnosis, chronic pain may be characterized as part of a somatoform disorder or a functional somatic syndrome (FSS) such as somatoform pain disorder or fibromyalgia syndrome (FMS) respectively. These disorders are characterized by distressing and functionally disabling somatic symptoms with chronic pain as the most frequent and clinically relevant complaint. This is also true for the multisomatoform disorder (MSD) [1, 2]. The diagnostic construct of MSD is used to acknowledge the common traits of these FSS subsets and to identify patients within different somatic and psychological specialities [2, 3].

MSD has a prevalence of 8% [3] and is defined by three or more medically unexplained, currently bothersome physical symptoms plus a long (more than 2 years) history of somatization. The pathophysiology of pain in MSD is not completely understood but both environmental and genetic factors, influencing allostatic systems [4] processing behavioral or physiological stressors, are considered. The importance of genetic influences, especially on diseases with chronic widespread pain as the main symptom, has been further investigated in a population-based twin study of FSS [5]. A large body of research has been devoted to the role of single-nucleotide polymorphisms (SNP) in genes relevant to pain physiology. Results are not consistent but suggest a role of SNPs in serotonergic and dopaminergic but not the COMT-genes in the etiology of MSD [6–8].

Both animal and epidemiological data show that adverse childhood experience (ACE) is a major risk factor for the development of FSS or a somatoform disorder [9–11]. Large population-based studies showed associations which strongly suggest common underlying mechanisms of different subsets of FSS [12]. It has been shown that environmental and biographical, especially ACE, are associated with many psychiatric and painful conditions [13, 14]. Higher degrees of childhood trauma have been associated with increased DNA methylation within the glucocorticoid promoter and consequently higher salivary cortisol levels after a laboratory stressor [15]. Therefore, we hypothesized that epigenetic regulation of pain-related genes is influenced by early life experiences and could be part of the underlying mechanism of patients with MSD experiencing chronic pain.

Sensation of pain requires the generation of action potentials for which nociceptive nerve endings express various receptor molecules which serve as a basis for selective signaling of different sensory qualities. Among these, members of the transient receptor potential (TRP) family of ion channels are the most widely studied, one of which is the transient receptor potential ankyrin 1 (TRPA1) receptor. TRPA1 has been shown to play a role in detecting cold pain, cold hypersensitivity, and irritants produced through tissue injury [16, 17]. TRPA1 may also be involved in mechanosensation [18–22], neurogenic inflammation, central sensitization, microglia activation, and transition from acute to chronic pain [18, 20, 21, 23–25]. In human trials, TRPA1 agonists applied to the skin indeed induce mechanical hypersensitivity [26] and heat hyperalgesia [26, 27].

Allodynia, mechanical and thermal hypersensitivity are abundant symptoms in patients with MSD, somatoform disorders, and FSS without the existence of a clear pathophysiological explanation. TRPA1 has been suggested as a possible mediator in these processes, as it has been shown to play a role in pathological pain states [28–30]. In addition to traditional SNP and point mutations, epigenetic mechanisms have been implicated in chronic pain states [31–33].

In a study of monozygotic twins as well as unrelated individuals, Bell et al. analyzed differentially methylated regions associated with high or low heat pain sensitivity. Of 5.2 million loci screened per person, they detected the strongest signal of association in the promoter region of TRPA1. The promoter region of TRPA1 was hypermethylated with low heat pain threshold indicating a role of TRPA1 in heat-induced pain [34].

Gombert et al. evaluated the methylation status of 47 single CpGs in the promoter sequence of TRPA1 in a trial of healthy volunteers undergoing evaluation of the individual pressure pain threshold through standardized algometry [35]. Hypermethylation of CpG -628 correlated significantly with low pressure pain thresholds, an effect more pronounced in women. With regards to transcription factor interaction, both Pax6 and Sp1 can exhibit positive and negative regulatory effects on gene expression through binding to CpG-rich sites and is affected by the methylation status of these regions [36]. Their role in the regulation of TRPA1 expression has not been studied at this point. Only Zavala et al. could demonstrate involvement of Sp1 in the expression of transient receptor potential vanilloid 1 (TRPV1) in dorsal root ganglia of rats [37, 38]. Due to its widespread occurrence and involvement in numerous regulatory processes, the meaning of this finding is not clear and further work is necessary to elucidate a potential role of Sp1 in regulating TRPA1 gene expression in health and disease.

The feasibility of using a questionnaire-based assessment of pain in conjunction with the analysis of DNA methylation levels has previously been demonstrated by Sukenaga et al. [39, 40]. The group observed a statistically significant correlation between an increase in mean methylation levels of the TRPA1 promotor and the number of neuropathic pain symptoms as measured by the DN4 questionnaire [39]. They also found TRPA1 mRNA levels to be inversely correlated with the number of pain symptoms observed [39, 40].

This would be in accordance with existing data showing that early childhood experience and environmental variables during early life stages influence methylation levels [41, 42]. In a study of 119 twin and 35 female pairs, Peng et al. found an association between methylation of five stress related genes and depression, accounting for approximately 20% of the association between childhood trauma and depression [43]. Similarly, clinical experience and research tell us that chronic pain states and pain intensity are aggravated by a history of traumatic events [13].

We therefore found it compelling to investigate the potential role of TRPA1 in patients with painful MSD and healthy volunteers in relation to childhood trauma. Building on previous evidence, we focused on the CpGs in the promoter region of TRPA1 that were shown to be associated with differences in heat pain as well as pressure pain sensitivity [34, 35].

## Materials and Methods

### Subjects

The current collective was previously investigated regarding the role of single-nucleotide polymorphisms (SNPs) of different genes [6–8]. The study group consisted of 149 healthy individuals and 151 MSD patients. Recruitment took place at the outpatient pain clinic of the Hannover Medical School, Hannover, Germany, and the Clinic for Psychosomatic Medicine and Psychotherapy of the Hannover Medical School. Patients from several fibromyalgia support groups were also recruited, with the recruitment process lasting over the course of 12 months. The majority of patients were undergoing regular treatment at both institutions. Records of exact distribution were not kept. At the same time, healthy age- and gender-matched participants with no physical pain were recruited as the control group. After pre-selection by expert clinicians ruling out severe psychiatric or somatic conditions, a complete clinical examination in addition to a basic assessment through psychometric questionnaires took place at the time of recruitment (SF-36, Childhood Trauma Questionnaire, Post-traumatic stress diagnostic scale). All patients presented with chronic widespread pain as the main symptom. Diagnosis of MSD was aided via the administration of the German version of the 36-item Short Form 36 (SF-36) questionnaire, i.e., the Physical Component Summary score needed to be ≤ 40, demonstrating strong psychophysiological strain. Additionally, to check for the presence of MSD, a modified interview of the somatoform disorders section of the Structured Clinical Interview for the Diagnostic and Statistical Manual of Mental Disorder IV (DSM-IV) (SCID) was used [1, 2, 6–8]. Further assessment was carried out through the Symptom Checklist 27 (SCL-27) [44], Patient Health Questionnaire (PHQ) [45], and Trier Inventory of Chronic Stress (TICS) [46]. Exclusion criteria were age younger than 18 years, insufficient German language skills, insufficient cognitive abilities, severe and chronic somatic diseases (e.g., severe heart failure, encephalitis disseminate, dementia), and severe comorbid mental disorder, causing major impairment of social functioning (e.g., schizophrenia, severe mood disorders, personality disorders, substance abuse).

Additionally, participants answered all 34 items of the Childhood Trauma Questionnaire (CTQ) on a five-point rating scale (1 = “not at all” to 5 = “very much”). The CTQ subscales describe emotional abuse, physical abuse, sexual abuse, emotional neglect, and physical neglect. Subscale scores are computed by summing up the score of the individual items. This results in a score with a range between 5 and 25 points. The resulting score is then categorically rated from no trauma to extreme trauma (1–4) for each subscale individually as previously reported [47, 48]. In order to differentiate between participants with severe multiple trauma events and mild or no trauma, we first binned the resulting subscale categories: none to mild trauma (≤ 2) and severe trauma (> 2) resulting in two scores (0 or 1). We then added these scores (possible summary result range: 0 to 5) and split the participants in three groups: no (0 points), mild (1–2 point), and severe (> 2 points) trauma. Blood samples were collected and used for DNA extraction, laboratory, and epigenetic analysis.

The study followed the guidelines of the revised UN Declaration of Helsinki in 2000 (Edinburgh, 52. General Meeting). Following approval by the ethics committee of the Hannover Medical School (study protocol number 4757), informed consent was obtained from all patients and controls for blood sampling, genotyping, and clinical measurements.

### Quantitative sensory testing

Quantitative sensory testing was developed by the German Research Collaborative for Neuropathic Pain (DFNS, “Deutscher Forschungsbund Neuropathischer Schmerz”) [49]. It consists of seven tests and 13 different parameters which cover all relevant submodalities of the somatosensory system. Of these, six tests covering 11 modalities were chosen for this study: cold detection threshold (CDT), warm detection threshold (WDT), thermal sensory limen (TSL), paradoxical heat sensations (PHS), cold pain threshold (CPT), heat pain threshold (HPT), mechanical detections threshold (MDT), mechanical pain threshold (MPT), wind-up-ratio (WUR), vibration detection threshold (VDT), and pressure pain threshold (PPT).

Thermal measurements were conducted using a Thermotester Typ TSA-II 2001 (MEDOC Ltd., Israel). For determining MDT, von Frey filaments (Opti-Hair2, MARSTOCK-nervtest, Marburg, Germany) were used. Evaluation of MPT was realized with standardized needle stimulators (Institute of Physiology and Pathophysiology, Mainz University, Germany). VDT was evaluated using a standardized 64Hz tuning fork with an 8/8 scale. PPT measurements were carried out using an Algometer Typ II, SOMEDIC, Sollentuna, Sweden) with a contact area of 1 cm^2^.

All test subjects were exposed to the same environmental variables: quiet room, room temperature between 21 and 23 °C, and no view of the computer screen or measuring scales used. The sequence of quantitative sensory testing (QST) measurements was identical for all subjects. Initially, the dorsum of the hand of the subject’s handedness was tested as the control area. The test area, defined as the most painful body area for patients and the paravertebral musculature at L4/5 on the side of the individual’s handedness for controls, followed. The QST test sequence lasted approximately 1 h.

### DNA isolation

Blood was collected from each subject using two 4 mL EDTA tubes that were then stored at − 80° until extraction. Genomic DNA from patients and controls was extracted using a standard high-salt extraction method. A small subset of DNA samples was isolated by using a commercially available DNA isolation kit (QiAmp® blood kit, Qiagen, Hilden, Germany) according to the manufacturer’s instructions.

### Determination of methylation rates

Analysis of the TRPA1 promoter region was performed as reported earlier [35]. The region of interest (base pairs -750 to -380 from the TSS of ENSEMBL gene # ENSG00000104321) containing the CpG hotspot previously identified in an epigenome-wide association study (CpG -628) [34]. Bisulfite conversion was performed using the Epitect conversion kit (Qiagen, Hilden, Germany) following manufacturer recommendations. The region was amplified using a PCR with bisulfite-DNA-specific primers following the protocols mentioned in the online supplements (Additional file 1: Figure S1). The amplicon was sequenced using a 3750 Capillary Sequencer from ABI (ABI Life Technologies, Grand Island, USA).

Sequence analysis and determination of methylation rates for each CpG site were conducted using the Epigenetic Sequencing Methylation analysis software [50]. The methylation rate of each CpG site per subject was estimated by determening the ratio between normalized peak values of cytosine and thymine.

### Quality control

We successfully measured other genes in this collective (unpublished data). Also, exactly the same analysis strategy was used for our previous publication on this topic (see Gombert et al.) [35]. The overall variance of measured results for TRPA1 and other genes was very low indicating a high level of precision of the collected data.

### Prediction of transcription factor binding sites

In order to analyze methylated sequences for potential binding of transcription factor (TFs), we predicted binding sites using Geneious 11 (Biomatters, Auckland, New Zealand) allowing for 1 mismatch base and confirmed the findings using the Alggen Promo tool (http://alggen.lsi.upc.es/cgi-bin/promo_v3/promo/promoinit.cgi?dirDB=TF_8.3) on the same sequence. Both tools access the freely available resources available through the Transfac public database (http://gene-regulation.com/cgi-bin/pub/databases/transfac/search.cgi) [51].

### Statistical analysis

**Fig. 1:**
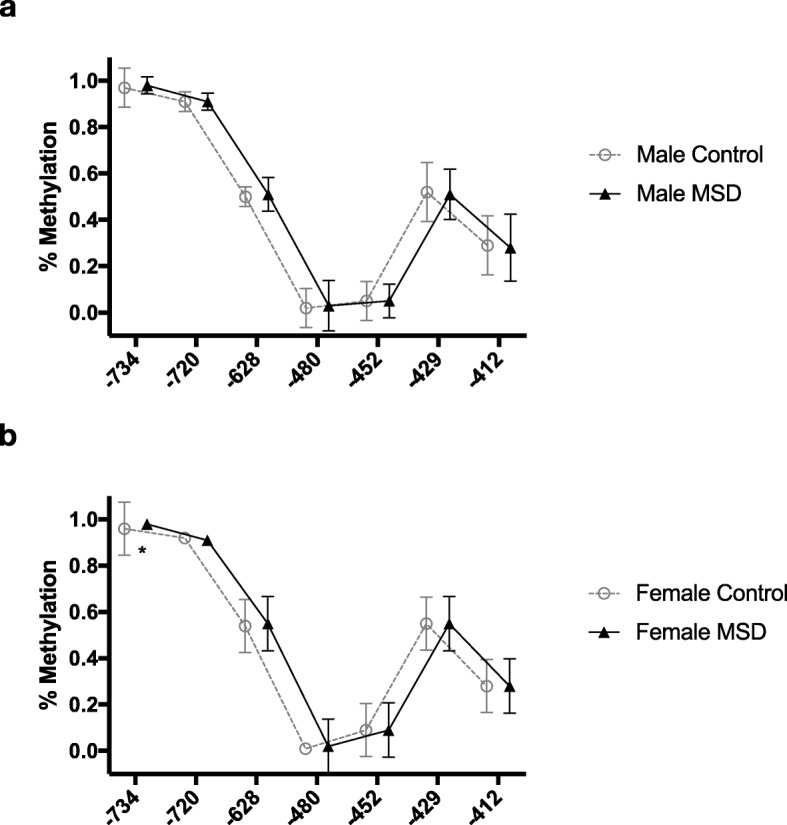
Methylation for each CpG position is depicted for each sex and control (gray circles) or multisomatoform disorder (MSD) cohort (filled triangles). In the entire population not divided by gender (**a**), men (**b**), and women (**c**), individual levels between cohorts reveal similar methylation. CpG position is noted in relation to transcription start site (TSS) according to ENSEMBL entry for the TrpA1 gene (#ENSG00000104321). Error bars depict standard deviation

Sequence quality was assessed via Sequence Scanner v1.0 software (ABI Life Technologies). Due to blood sample quality (inclusion of all samples with more than 95% valid measurements), 15 patient and four control samples had to be excluded. All statistical calculations were performed using the Statistical Package for the Social Sciences (SPSS, IBM, Armonk, NY). We used GraphPad Prism for Windows 5.03 for data illustration (Graphpad Software Inc, La Jolla, CA). All CpG sites were sequenced successfully and could be included in the analysis. Methylation levels for individual CpG sites are provided in Fig. 1. Deviance from normal distribution was checked according to Shapiro-Wilk. In case of normally distributed variables, parametric methods were used; for all other cases, nonparametric tests were used. Spearman correlations were used to investigate associations of methylation levels and QST measurements in patients. We calculated mixed linear models to detect significant fixed effects of different variables on methylation rate. *P* values were corrected using the Bonferroni correction method. Multiple linear regression (stepwise method) was conducted to identify significant predictors for pressure pain threshold in female controls. In each analysis, a *p* value of ≤ 0.05 was considered significant. One-way ANOVA was used to check for differences in methylation levels between patients and controls after grouping for childhood trauma levels. Two-way ANOVA was used to investigate possible interaction effects of MSD and childhood trauma on methylation levels. Sequential mediation analysis was performed to assess possible mediating influences of childhood traumatization and methylation levels on the different QST measurements observed in patients with MSD. Calculations were performed using the Process 3.3 macro for SPSS by A. Hayes [52]**.**

## Results

### Demographic data

All patients fulfilled the diagnostic criteria for MSD according to the Diagnostic and Statistical Manual of Mental Disorder-IV (DSM-IV). Both groups showed no significant differences regarding gender and age (*p* > 0.05) (control group: mean age, 52.1 ± 9.9 years; 73% women and 27% men; MSD group: mean age, 54.4 ± 10.1 years; 82% women and 18% men), whereas the physical component summary score of the SF-36 showed a significant difference between patients and controls (*p* < 0.001) (Table 1). Complete QST data was obtained in all patients but only in 140 of the 149 control subjects. Due to sample quality, the epigenetic analysis could not be performed in four controls and 15 patients. The final sample was made up of 136 patients (123 female/13 male) and 145 controls (127 female/18 male).

**Table 1:** Group characteristics and methylation levels

	Control group				MSD patients			
	Male		Female		Male		Female	
	Mean	Standard deviation	Mean	Standard deviation	Mean	Standard deviation	Mean	Standard deviation
Age (years)	46.06	14.44	53.32	8.72	51.67	9.38	55.14	10.11
Body mass index	25.02	3.38	24.20	3.96	26.63	3.75	27.02	5.26
SF-36 physical sum score	55.11	3.57	53.86	5.95	30.09	7.75	28.62	7.83
VAS (0–10)	1.17	2.27	1.82	2.62	7.12	1.50	7.00	1.55
CpG -734 methylation	0.97	0.07	0.96	0.09	0.98	0.04	0.98	0.05
CpG -720 methylation	0.91	0.03	0.92	0.03	0.91	0.04	0.91	0.03
CpG -628 methylation	0.50	0.06	0.54	0.06	0.51	0.07	0.55	0.06
CpG -480 methylation	0.02	0.07	0.01	0.03	0.03	0.10	0.02	0.06
CpG -452 methylation	0.05	0.07	0.09	0.08	0.05	0.05	0.09	0.11
CpG -429 methylation	0.52	0.11	0.55	0.08	0.51	0.09	0.55	0.09
CpG -412 methylation	0.29	0.13	0.28	0.07	0.28	0.13	0.28	0.09
Mean methylation	0.47	0.03	0.48	0.03	0.47	0.04	0.48	0.04

Group characteristics, psychometric scores, pain measured on visual analog scale (VAS) and methylation levels of individual CpGs and mean methylation (range 0–1.0, i.e., no to complete methylation) of MSD patients and controls split according to gender

When comparing the methylation levels of the investigated CpGs within the TRPA1 promoter region between MSD patients and healthy controls, no significant differences were found (Fig. 1a). As methylation levels are known to be gender-dependent [53] and MSD has a significantly higher prevalence in females, we decided to perform further analyses according to gender. Methylation levels showed no significant differences between male patients and male controls (Fig. 1b). Comparing female patients to female controls revealed a significant difference in methylation levels at CpG -734 (Fig. 1c). However, we discovered that this result is mainly driven by outliers as most samples showed a methylation rate of 100% at CpG -734 in patients as well as healthy controls. Further investigation focused mainly on female study participants as the small sample size of male participants provides insufficient explanatory power. Significant findings in female participants were checked in the male population (Additional file 2: Figure S2 and Additional: file 3: Table S1).

### Correlation between methylation rate, quantitative sensory testing, and psychometric data

**Fig. 2:**
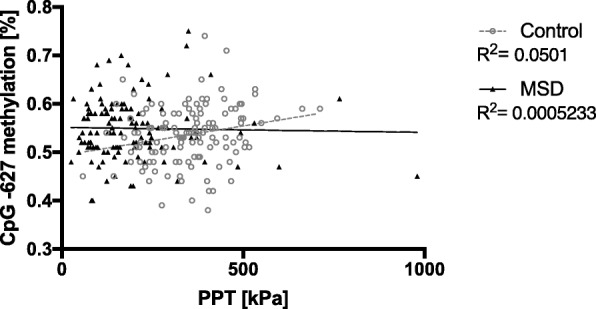
Mean methylation of CpG -628 is plotted against pressure pain threshold (PPT) (kPa) for female controls and MSD patients. While correlation differs between cohorts, predictability, estimated by *R*^2^ values for the linear function, is 5% in controls and 0.05% in MSD patients

We calculated Spearman correlation coefficients for the seven individual CpG sites and checked their correlations with the QST data and psychometric results. In female controls, this analysis revealed significant results for the correlation of CpG -628 (*p* = 0.012, rs = 0.227) with pressure pain threshold (ppt) (see Fig. 2 and Additional file 3: Table S1) giving a corrected *R*^2^ of 0.0510 meaning that 5% of variance in PPT is explained by difference in methylation levels. This is in keeping with previous studies [34, 35]. There was also a correlation of CpG -412 with the mechanical pain threshold (MPT) (*p* = 0.035, rs = − 0.191). In male controls, we observed the following correlations: CpG -628 (*p* = 0.018, rs = − 0.622) with pressure pain threshold (PPT) (Additional file 2: Figure S2 and Additional file 4:Document S1), as well as CpG -412 with mechanical pain threshold (MPT) (*p* = 0.038, rs = 0.579). To further investigate potential statistical relationships, we performed stepwise linear regression analysis including age, BMI, mean methylation, and methylation at the individual CpG sites as predictors and pressure pain threshold as the dependent variable. We found the best fitting model to include CpG -628, -429, and -412 (*R*^2^ = 0.118, *R*^2^_corr_ = 0.094, *F*_(2)_ = 4.493, *p* = 0.003) showing only a weak ability (9,4%) to account for the variance in pressure pain threshold. No such correlation was found in female patients.

However, in female patients CpG -429 (*p* = 0.02, rs = − 0.222) and mean methylation (*p* = 0.014, rs = − 0.235) showed significant negative correlation with reported VAS pain scores while CpG -628 methylation trended toward a significant correlation (*p* = 0.063, rs = − 0.179). Additionally, the physical pain component of the SF-36 questionnaire demonstrated significant correlation with CpG -628 methylation (*p* = 0.034, rs = 0.200), i.e., higher methylation levels were associated with less experience of painful symptoms.

To investigate a possible influence of psychological variables on methylation status, we further calculated correlation coefficients for CTQ scores, SCL-27, TICS scores, and PHQ scores. We found significant correlations of CpG -628 (*p* = 0.023, rs = − 0.215), CpG -429 (*p* = 0.015, rs = − 0.231), CpG -480 (*p* = 0.001, rs = − 0.305), and mean methylation (*p* = 0.004, rs = − 0.274) with cumulative CTQ scores in female patients, i.e., higher scores indicating childhood trauma were correlated with decreased methylation.

Since both CpG -480 and -429 show similar positive correlations and both are functionally positioned in the predicted binding motif of the transcription factor Sp1, we decided to average the methylation effect on these positions, assuming a similar effect on expression. We found averaged methylation rates of the two CpGs to have a higher degree of correlation with cumulative CTQ scores (*p* = 0.001, rs = − 0.305) than the individual CpGs. Most CTQ subscores correlated significantly as well (see Additional file 3: Table S1).

**Fig. 3:**
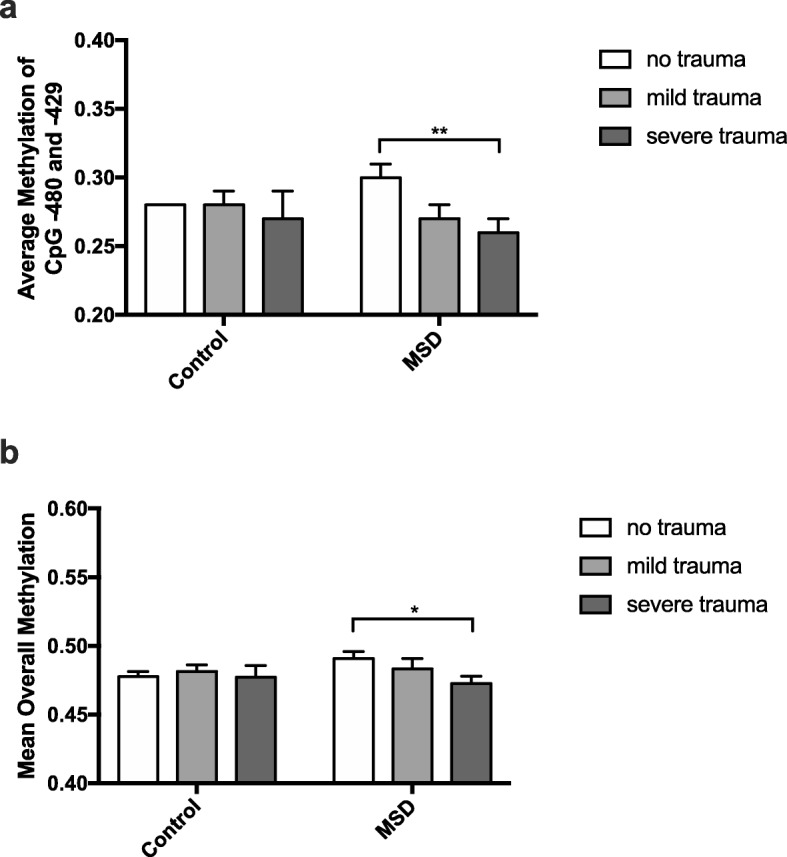
**a** Mean methylation of average CpG methylation of CpG -480 and -429 is displayed for females from control and MSD cohort according to the CTQ severity score. Non-parametrical testing of the three groups reveals significant differences between female patients with severe trauma and mild trauma as well as severe trauma and no trauma. After correction for multiple comparisons, patients with severe trauma significantly differ from patients without trauma (*p* = 0.01, test statistic = 21.107, df = 2). **b** Overall mean methylation of female patients and controls according to CTQ severity score. Non-parametric testing shows a significant difference in mean methylation overall between patients with “no trauma” and “severe trauma” (*p* = 0.012) which remained significant after correcting for multiple comparisons

Dividing female patients into groups according to severity of childhood trauma as described above, we used Kruskal-Wallis tests for ascertaining between group differences of combined average methylation of CpGs -480 and -429 as well as overall mean methylation. Average methylation at CpGs -480 and -429 showed significant differences between “no trauma” and “severe trauma” (*p* = 0.003, test statistic = 21.107, std.error = 7.211), as well as “no trauma” and “mild trauma” (*p* = 0.031, test statistic = 16.392, std.error = 7.589) in the MSD group. (Fig. 3a). After correction for multiple comparisons only the difference between “no trauma” and “severe trauma” groups remained significant (*p* = 0.01 test statistic = 21.107, std.error = 7.211). There was also a significant difference in overall mean methylation between “no trauma” and “severe trauma” (*p* = 0.012, test statistic = 18,116, std.error = 7217) which remained significant after correcting for multiple comparisons (Fig. 3b).

In a two-way ANOVA analysis, no significant interaction was observed between being diagnosed with MSD and level of childhood trauma on methylation levels (mean methylation (*F* (2, 225) = 1.01, *p* = 0.37) and average methylation at CpGs -480 and -429(*F* (2, 225) = 1.86, *p* = 0.16)). Main effects tests showed a significant group difference between “no trauma” and “severe trauma” in female patients (*p* = 0.008) with regards to average methylation at CpGs -480 and -429; the initially observed significance for mean methylation levels between “no trauma” and “mild trauma” groups was lost after adjusting for multiple comparison. Since the interaction between trauma and MSD appears not significant in our results, this would suggest that the interaction between trauma and MSD is not the driving factor for methylation changes.

Due to the significant methylation differences between trauma groups and correlation between methylation levels and cumulative CTQ scores, we decided on cumulative CTQ scores, mean methylation, and average methylation at functional CpGs -480 and -429 as likely mediators for altered sensory profiles in MSD. We conducted serial mediation analysis to investigate their possible mediation effects on the influence of MSD on those QST measurements known to significantly differ between patients and controls.

We found mediation effects of cumulative CTQ scores and mean methylation on the effect of a diagnosis of MSD on mechanical pain threshold at the test site (indirect effect = .69, SE = .54, 95% CI [0.01, 2.06]) and tactile perception threshold at the control (indirect effect = .03, SE = .02, 95% CI [0.01, 0.06]) as well as the test site (indirect effect = .15, SE = .12, 95% CI [0.001, 0.45]). Additionally, we found a mediation effect of cumulative CTQ scores on the effect that a diagnosis of MSD exhibits on pressure pain threshold (indirect effect = 2.72, SE = 1.60, 95% CI [0.015, 6.28]). Interestingly, the overall model of the influence of MSD on sensory profiles, cumulative CTQ score, and mean methylation was non-significant with regards to mechanical pain threshold. However, this is not a necessary requirement for mediation to occur [54]. For complete mediation analysis, see Additional file 5: Document S2.

## Discussion

TRP channels are essential for sensing various painful stimuli of different modalities. Patients with MSD experience more pain, more often and from lesser events than other patients without there being a clear pathophysiological explanation. One possible avenue of investigation leads toward TRP receptors, especially TRPA1 and its regulation through epigenetic mechanisms.

In our study, we decided to focus on female patients and controls as MSD has a known higher prevalence in women and because epigenome-wide association studies have demonstrated autosomal differences in methylation patterns between women and men [53]. We performed a methylation analysis of seven CpGs in the region of the TRPA1 core promoter that revealed differing methylation levels at individual CpG sites. Our findings demonstrate the same significant correlation between CpG -628 and pain thresholds at the control site (Fig. 2) as previously demonstrated [34, 35] in addition to a significant correlation between CpG -412 and pressure pain threshold at the test site of healthy female controls.

In contrast, no correlation between individual CpGs as well as mean methylation and pressure pain threshold could be observed compared to healthy controls. This could be due to abolished regulatory mechanisms of TRPA1 expression or other non-mechanistic factors having a more pronounced effect on pain sensitivity.

Our hypothesis is that CTQ-driven methylation changes alter the function of one of the potential contributors to pressure pain, ultimately leading to an increased likelihood of the MSD diagnosis due to chronic pain. Mediation analysis supports this hypothesis, as mediation effects of mean methylation and CTQ score on mechanical pain threshold as well as averaged methylation of the functionally related CpGs -480/-429 and CTQ scores on pain pressure threshold were observed. Since both parameters are connected to the MSD phenotype, our model might be one explanation for the interconnection of epigenetic readouts that are both linked to traumatic childhood events and probably contribute to functional dysregulation of pain receptor expression. While both the connection of CTQ to altered methylation [41–43] and the potential modulatory effect of TRPA1 methylation on expression (Gombert et al.) support this mechanism, there is no indication concerning cause and effect. Future studies with longitudinal character will provide insight into this important aspect.

In addition, as correlation coefficients are low in our data which is in keeping with data published by Gombert and Bell, a definitive answer regarding the direction of correlation cannot be given at this moment [34, 35].

Observing correlation between CTQ subscores and TRPA1 methylation, we calculated a severity score to easily differentiate between different levels of trauma as described previously [48]. Additional analysis revealed significant differences in average combined methylation of the functionally similar CpG -429 and CpG -480 as well as overall mean methylation between female patients with no and severe childhood trauma. No such differences were found in controls. In spite of this finding, two-way ANOVA analysis investigating a possible interaction between MSD and degree of childhood trauma revealed no interaction between presence of MSD and level of childhood traumatization.

A limitation of both our, as well as all studies by Gombert, Bell and Sukenaga, is the utilization of DNA from white blood cells for analysis of methylation levels. While it has been demonstrated that methylation levels are similar in different tissues [55], cases of DNA methylation being tissue-specific have also been reported [56]. Neuronal tissue would have been preferable but is not readily available in most study designs. A further limitation is the lack of data regarding potential participants who declined to take part after positive suitability screening as well as lack of data concerning the recruitment location (support group, Pain Clinic, Department of Psychosomatics and Psychotherapy). This might lead to a degree of self-selection bias. This is mitigated, however, by the stringent selection process that resulted in a study population with a high disease burden.

## Conclusion

To our knowledge, the present study is the first to thoroughly characterize a large collective of patients with MSD and chronic pain as the leading symptom and a group of age- and gender-matched controls using various psychometric questionnaires and extensive quantitative sensory testing. Our study provides further evidence of TRPA1 promoter methylation playing a role in pain regulation in healthy volunteers as well as in patients suffering from chronic pain states. These findings were further expanded by the influence of childhood trauma on methylation levels in the studied patient population. However, our data is purely observational, and it will be interesting to see how TRPA1 promotor methylation changes over time in response to different interventions. Prospective longitudinal studies are necessary to further evaluate the role of TRPA1 and its promoter methylation in the pathogenesis of chronic widespread pain.

## Additional files

Additional file 1: **Figure S1.** Mean methylation of CpG -628 is plotted against PPT (kPa) for male controls and MSD patients. While correlation differs between cohorts, predictability, estimated by *R*^2^ values for the linear function, is 5% in controls and 0.05% in MSD patients (PDF 33 kb)

Additional file 2: **Figure S2.** A. Mean methylation of average CpG methylation of CpG -480 and -429 is displayed for males from control and MSD cohort according to the CTQ severity score. Non-parametrical testing of the three groups revx`ealed no significant differences. B. Overall mean methylation of male patients and controls according to CTQ severity score. Non-parametric testing showed no significant difference in mean methylation overall between patients with “no trauma” and “severe trauma” (PDF 34 kb)

Additional file 3: **Table S1.** Spearman correlations for selected variables (XLSX 17 kb)

Additional file 4: **Document S1.** Sequencing Primers and PCR program for the TRPA1 Promoter (DOCX 14 kb)

Additional file 5: Complete mediation analysis data. (DOCX 58 kb)

## Data Availability

The datasets used and/or analyzed during the current study are available from the corresponding author on reasonable request.

## Abbreviations

ACE: Adverse childhood experience
CDT: Cold detection threshold
COMT: Catecholamine-O-methyltransferase
CpG: Cytosin-phosphate-Guanine
CPT: Cold pain threshold
CTQ: Childhood Trauma Questionnaire
DSM-IV: Diagnostic and Statistical Manual of Mental Disorder IV
FMS: Fibromyalgia syndrome
FSS: Functional somatic syndrome
HPT: Heat pain threshold
MDT: Mechanical detection threshold
MPT: Mechanical pain threshold
MSD: Multisomatoform disorder
PHQ: Patient Health Questionnaire
PHS: Paradoxical heat sensations
PPT: Pressure pain threshold
QST: Quantitative sensory testing
SCID: Structured clinical interview
SCL-27: Symptom Checklist 27
SF-36: Short Form 36
SNP: Single-nucleotide polymorphism
TF: Transcription factor
TICS: Trier Inventory of Chronic Stress
TRPA1: Transient receptor potential ankyrin 1
TRPV1: Transient receptor potential vanilloid 1
TSL: Thermal sensory limen
VDT: Vibration detection threshold
WDT: Warm detection threshold
WUR: Wind-up ratio
