# Supplementary figures and images for: Childhood traumatization is associated with differences in TRPA1 promoter methylation in female patients with multisomatoform disorder with pain as the leading bodily symptom
Source: Clin Epigenetics. 2019 Aug 28;11:126. doi: 10.1186/s13148-019-0731-0 (PMC6712620; doi:10.1186/s13148-019-0731-0)

# Figure S1

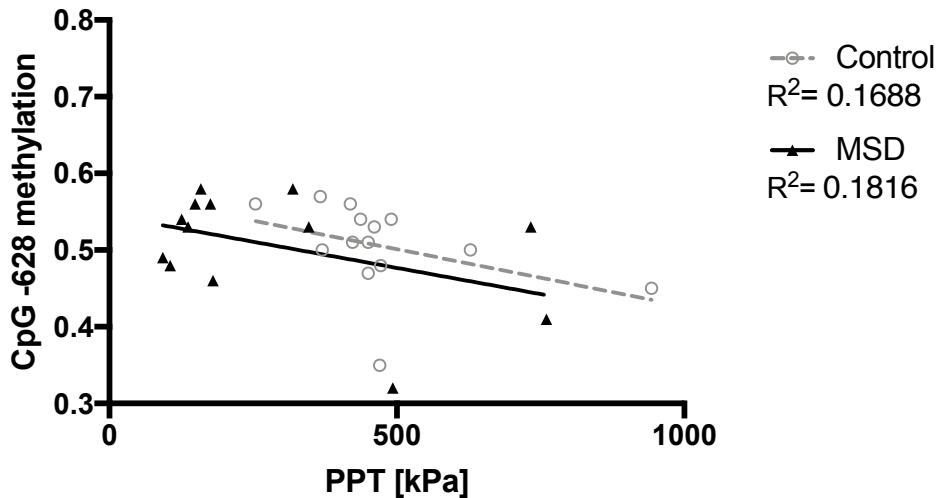

Supplement: Supplementary file 1 — Figure S1. Mean methylation of CpG -628 is plotted against PPT (kPa) for male controls and MSD patients. While correlation differs between cohorts, predictability, estimated by R2 values for the linear function, is 5% in controls and 0.05% in MSD patients (PDF 33 kb) [file 13148_2019_731_MOESM1_ESM.pdf]

Figure S2

a

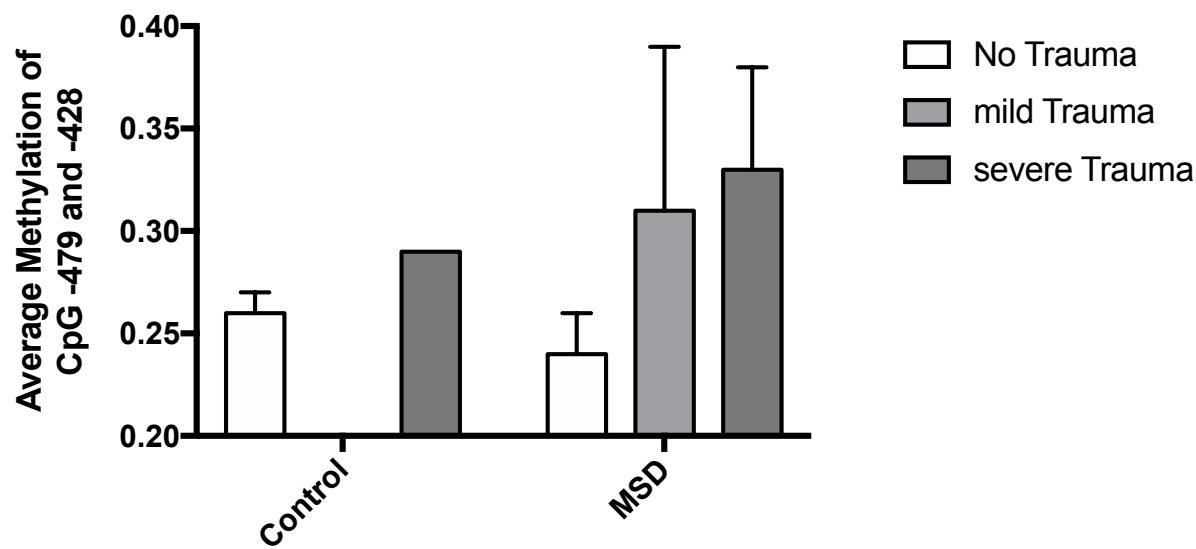

b

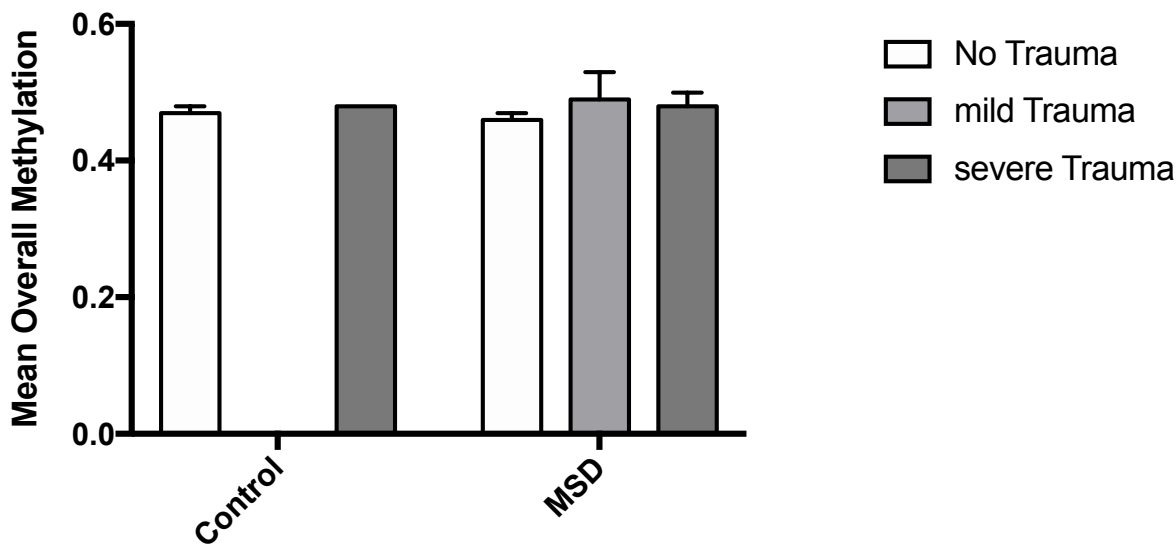

Supplement: Supplementary file 2 — Figure S2. A. Mean methylation of average CpG methylation of CpG -480 and -429 is displayed for males from control and MSD cohort according to the CTQ severity score. Non-parametrical testing of the three groups revx`ealed no significant differences. B. Overall mean methylation of male patients and controls according to CTQ severity score. Non-parametric testing showed no significant difference in mean methylation overall between patients with “no trauma” and “severe trauma” (PDF 34 kb) [file 13148_2019_731_MOESM2_ESM.pdf]
