# Supplementary material for: Childhood traumatization is associated with differences in TRPA1 promoter methylation in female patients with multisomatoform disorder with pain as the leading bodily symptom
Source: Clin Epigenetics. 2019 Aug 28;11:126. doi: 10.1186/s13148-019-0731-0 (PMC6712620; doi:10.1186/s13148-019-0731-0)
Supplement: Supplementary file 4 — Document S1. Sequencing Primers and PCR program for the TRPA1 Promoter (DOCX 14 kb) [file 13148_2019_731_MOESM4_ESM.docx]

**Sequencing Primers and PCR program for the TRPA1 Promoter**

| Primer name | Primer Sequence | Tm |
| --- | --- | --- |
| F1 | GTTTGTATTAGATAGTTTTTTTGTTTG | 55° |
| R1 | TCCTACAAACCTATATTTCCCAC | 55° |
| Seq (F1) | GTTTGTATTAGATAGTTTTTTTGTTTG | 55° |

**PCR**

Primers: F1 and R1

Program: Touchdown PCR starting at 65°

| 15 min | 95°C |  |
| --- | --- | --- |
| 1 min | 97°C |  |
| 45 sec | 95°C | 15 x (-1°C per cycle) |
| 45 sec | 65°C |  |
| 1 min | 72°C |  |
| 45 sec | 95°C | 30 x |
| 45 sec | 50°C |  |
| 45 sec | 72°C |  |
| 5 min | 72°C |  |
| ∞ | 12°C |  |

**SeqPCR**

Primer: Seq (F1)

Program: SeqSTD

| 1 min | 96°C |  |
| --- | --- | --- |
| 10 sec | 96°C | 25 x |
| 5 sec | 50°C |  |
| 4 min | 60°C |  |
| ∞ | 12°C |  |
