# Supplementary material for: Childhood traumatization is associated with differences in TRPA1 promoter methylation in female patients with multisomatoform disorder with pain as the leading bodily symptom
Source: Clin Epigenetics. 2019 Aug 28;11:126. doi: 10.1186/s13148-019-0731-0 (PMC6712620; doi:10.1186/s13148-019-0731-0)
Supplement: Supplementary file 5 — Complete mediation analysis data. (DOCX 58 kb) [file 13148_2019_731_MOESM5_ESM.docx]

Document S2

[Annotated Result Summaries 2](#_Toc15468209)

[Original Output 4](#_Toc15468210)

[Mean methylation 4](#_Toc15468211)

[Mechanical pain threshold control site 4](#_Toc15468212)

[Mechanical pain threshold test site 6](#_Toc15468213)

[Tactile control threshold control site 9](#_Toc15468214)

[Tactile control threshold test site 12](#_Toc15468215)

[Pressure pain threshold control site 14](#_Toc15468216)

[Pressure pain threshold test site 17](#_Toc15468217)

[Mean methylation of CpG -480/-429 19](#_Toc15468218)

[Mechanical pain threshold control site 19](#_Toc15468219)

[Mechanical pain threshold test site 22](#_Toc15468220)

[Tactile control threshold control site 24](#_Toc15468221)

[Tactile control threshold test site 27](#_Toc15468222)

[Pressure pain threshold control site / methylation CpG -480/-429 29](#_Toc15468223)

[Pressure pain threshold test site 32](#_Toc15468224)

[CpG -628 (Bell / Gombert) 35](#_Toc15468225)

[Mechanical pain threshold control site 35](#_Toc15468226)

[Mechanical pain threshold test site 37](#_Toc15468227)

[Tactile control threshold control site 40](#_Toc15468228)

[Tactile control threshold test site 42](#_Toc15468229)

[Pressure pain threshold control site 45](#_Toc15468230)

[Pressure pain threshold test site 47](#_Toc15468231)

**Legend for Original ouput:**

**- Kontroll = Control or MSD**

**- QST08T = Mechanical pain threshold test site**

**- QST08K = Mechanical pain threshold control site**

**- QST07T = tactile detection threshold test site**

**- QST07K = tactile detection threshold control site**

**- QST13T = pressure pain threshold test site**

**- QST13K = pressure pain threshold control site**

**- CTQ1ges = CTQ total score**

**- mean_meth = mean methylation**

**- m2521_25 = mean methylation of CpG -480/-429**

**- m2373 = CpG -628 (Bell / Gombert)**

# Annotated Result Summaries

***Influence of MSD on Mechanical pain threshold via CTQ score and mean methylation***

MSD diagnoses predicts cumulative CTQ score b=10.22, t(224)=5,00, p<0.001

The overall model when performing regression on mean methylation with CTQ score and MSD Diagnosis as predictors is significant F(2, 223)=4.22, p=0.16, R2=.04

MSD diagnoses does not predict mean methylation b=0.01, t(223)=1.93, p=0.06

Cumulative CTQ score predicts mean methylation b=-0.0004, t(223)=-2.71, p=0.01

MSD does not predict mechanical pain threshold b=1.41, t(222)=.27, p=.79

Mean Methylation does not predict mechanical pain threshold b=-0.0004, t(222)=-2.71, p=0.01. Cumulative CTQ score does not predict mechanical pain threshold b=-.53, t(222)=-2.68, p=0.79. The total effect model was not significant F(1,224)=.0002, p=.99, R2=0.00 However, this is not a necessary requirement for mediation to occur. {Zhao et al., 2010, #97699}. Cumulative CTQ Score and mean methylation mediated the effect of MSD on mechanical pain threshold, indirect effect=.69, SE=.54, 95% CI[0.01, 2.06]

***Influence of MSD on tactile detection threshold at control site via CTQ score and mean methylation***

*Overall model of the influence of MSD, cumulative CTQ score and mean methylation on tactile detection threshold at the control site is significant F(3, 224)=4.52, p<0.01, R2=.09*

*MSD diagnoses does predict tactile detection threshold b=1.38, t(224)=3.24, p=0.001.*

*Cumulative CTQ score does not predict tactile detection threshold b=-0.02, t(224)=1.38, p=0.27.*

*Mean methylation does not predict tactile detection threshold b=-24.44, t(224)=-1.96, p=0.051.*

*In the total effect model MSD diagnoses does predict tactile detection threshold b=1.06, t(226)=2.36, p=0.02.*

*Cumulative CTQ Score and mean methylation mediated the effect of MSD on tactile detection threshold, indirect effect=.03, SE=.02, 95% CI[0.01, 0.06]*

***Influence of MSD on tactile detection threshold at test site via CTQ score and mean methylation***

Overall model of the influence of MSD, cumulative CTQ score and mean methylation is significant F(3, 224)=3.54, p=0.02, R2=.06

MSD diagnoses does predict tactile detection threshold b=6.01, t(224)=3.18, p=0.01.

Cumulative CTQ score does not predict tactile detection threshold b=0.02, t(224)=.22, p=0.83.

Mean methylation does not predict tactile detection threshold b=-39.13, t(224)=-1.66, p=0.10.

In the total effect model MSD diagnoses does predict tactile detection threshold b=5.97, t(226)=3.20, p=0.002.

Cumulative CTQ Score and mean methylation mediated the effect of MSD on tactile detection threshold, indirect effect=.15, SE=.12, 95% CI[0.001, 0.45]

***Influence of MSD on pressure pain threshold via CTQ score average methylation at CpGs -480 and -429***

The overall model when performing regression on average methylation at CpGs -480 and -429 with CTQ score and MSD Diagnosis as predictors is significant F(2, 224)=8.15, p=0.0004, R2=.08

MSD diagnoses does not predict average methylation at CpGs -480 and -429 b=0.01, t(224)=1.41, p=0.16

Cumulative CTQ score predicts average methylation at CpGs -480 and -429 b=-0.0009, t(224)=-3.9, p=0.001

Overall model of the influence of MSD, cumulative CTQ score and average methylation at CpGs -480 and -429 is not significant F(3, 223)=35.71, p<0.0001, R2=.28

MSD diagnoses does predict pressure pain threshold b=-155.37, t(223)=-8.96, p<0.001.

Cumulative CTQ score does not predict pressure pain threshold b=-0.07, t(223)=-0.10, p=0.91.

Average methylation at CpGs -480 and -429 does not predict pressure pain threshold b=-297,89 t(223)=165.45, p=0.073.

In the total effect model MSD diagnoses does predict pressure pain threshold b=-156.41, t(225)=-9.13, p<0.001.

Cumulative CTQ Score and Average methylation at CpGs -480 and -429 mediated the effect of MSD pressure pain threshold, indirect effect=2.72, SE=1.60, 95% CI[0.015, 6.28]

# **Original Output**

# Mean methylation

## Mechanical pain threshold control site

Run MATRIX procedure:

*************** PROCESS Procedure for SPSS Version 3.3 *******************

Written by Andrew F. Hayes, Ph.D. www.afhayes.com

Documentation available in Hayes (2018). www.guilford.com/p/hayes3

**************************************************************************

Model : 6

Y : QST08K

X : Kontroll

M1 : ctq1ges

M2 : mean_met

Sample

Size: 227

Custom

Seed: 1

**************************************************************************

OUTCOME VARIABLE:

ctq1ges

Model Summary

R R-sq MSE F(HC3) df1 df2 p

,3190 ,1017 226,1670 24,5207 1,0000 225,0000 ,0000

Model

coeff se(HC3) t p LLCI ULCI

constant 35,8654 1,0184 35,2182 ,0000 33,8586 37,8722

Kontroll 10,0823 2,0361 4,9518 ,0000 6,0701 14,0946

**************************************************************************

OUTCOME VARIABLE:

mean_met

Model Summary

R R-sq MSE F(HC3) df1 df2 p

,1839 ,0338 ,0012 4,0711 2,0000 224,0000 ,0183

Model

coeff se(HC3) t p LLCI ULCI

constant ,4920 ,0064 76,8747 ,0000 ,4794 ,5046

Kontroll ,0092 ,0049 1,8789 ,0616 -,0004 ,0188

ctq1ges -,0004 ,0001 -2,6658 ,0082 -,0007 -,0001

**************************************************************************

OUTCOME VARIABLE:

QST08K

Model Summary

R R-sq MSE F(HC3) df1 df2 p

,1143 ,0131 2850,1998 1,2057 3,0000 223,0000 ,3085

Model

coeff se(HC3) t p LLCI ULCI

constant 92,7769 43,3431 2,1405 ,0334 7,3623 178,1914

Kontroll -11,4506 7,2109 -1,5880 ,1137 -25,6609 2,7597

ctq1ges ,0933 ,2385 ,3913 ,6960 -,3767 ,5633

mean_met -63,2387 84,8339 -,7454 ,4568 -230,4174 103,9401

************************** TOTAL EFFECT MODEL ****************************

OUTCOME VARIABLE:

QST08K

Model Summary

R R-sq MSE F(HC3) df1 df2 p

,1017 ,0104 2832,6560 2,3655 1,0000 225,0000 ,1254

Model

coeff se(HC3) t p LLCI ULCI

constant 65,8864 5,4508 12,0875 ,0000 55,1453 76,6275

Kontroll -10,8434 7,0502 -1,5380 ,1254 -24,7363 3,0495

************** TOTAL, DIRECT, AND INDIRECT EFFECTS OF X ON Y **************

Total effect of X on Y

Effect se(HC3) t p LLCI ULCI c_ps

-10,8434 7,0502 -1,5380 ,1254 -24,7363 3,0495 -,2031

Direct effect of X on Y

Effect se(HC3) t p LLCI ULCI c'_ps

-11,4506 7,2109 -1,5880 ,1137 -25,6609 2,7597 -,2145

Indirect effect(s) of X on Y:

Effect BootSE BootLLCI BootULCI

TOTAL ,6072 2,5567 -3,8465 6,1389

Ind1 ,9409 2,4065 -3,2676 6,2671

Ind2 -,5799 ,8715 -2,5280 1,0561

Ind3 ,2463 ,3671 -,4373 1,0694

Partially standardized indirect effect(s) of X on Y:

Effect BootSE BootLLCI BootULCI

TOTAL ,0114 ,0484 -,0729 ,1178

Ind1 ,0176 ,0456 -,0610 ,1205

Ind2 -,0109 ,0166 -,0486 ,0195

Ind3 ,0046 ,0070 -,0082 ,0205

Indirect effect key:

Ind1 Kontroll -> ctq1ges -> QST08K

Ind2 Kontroll -> mean_met -> QST08K

Ind3 Kontroll -> ctq1ges -> mean_met -> QST08K

*********************** ANALYSIS NOTES AND ERRORS ************************

Level of confidence for all confidence intervals in output:

95,0000

Number of bootstrap samples for percentile bootstrap confidence intervals:

10000

NOTE: A heteroscedasticity consistent standard error and covariance matrix estimator was used.

NOTE: Variables names longer than eight characters can produce incorrect output.

Shorter variable names are recommended.

------ END MATRIX -----

## Mechanical pain threshold test site

Run MATRIX procedure:

*************** PROCESS Procedure for SPSS Version 3.3 *******************

Written by Andrew F. Hayes, Ph.D. www.afhayes.com

Documentation available in Hayes (2018). www.guilford.com/p/hayes3

**************************************************************************

Model : 6

Y : QST08T mechanical pain threshold test site

X : Kontroll

M1 : ctq1ges

M2 : mean_met

Sample

Size: 226

Custom

Seed: 1

**************************************************************************

OUTCOME VARIABLE:

ctq1ges

Model Summary

R R-sq MSE F(HC3) df1 df2 p

,3228 ,1042 226,1701 24,9605 1,0000 224,0000 ,0000

Model

coeff se(HC3) t p LLCI ULCI

constant 35,8654 1,0184 35,2182 ,0000 33,8586 37,8722

Kontroll 10,2195 2,0455 4,9960 ,0000 6,1886 14,2504

MSD diagnoses predicts cumulative CTQ score b=10.22, t(224)=5,00, p<0.001

**************************************************************************

OUTCOME VARIABLE:

mean_met

Model Summary

R R-sq MSE F(HC3) df1 df2 p

,1881 ,0354 ,0012 4,2219 2,0000 223,0000 ,0159

Model

coeff se(HC3) t p LLCI ULCI

constant ,4923 ,0064 76,6898 ,0000 ,4796 ,5049

Kontroll ,0095 ,0049 1,9366 ,0541 -,0002 ,0192

ctq1ges -,0004 ,0001 -2,7109 ,0072 -,0007 -,0001

The overall model is significant F(2, 223)=4.22, p=0.16, R2=.04

MSD diagnoses does not predict mean methylation b=0.01, t(223)=1.93, p=0.06

Cumulative CTQ score predicts mean methylation b=-0.0004, t(223)=-2.71, p=0.01

**************************************************************************

OUTCOME VARIABLE:

QST08T mechanical pain threshold

Model Summary

R R-sq MSE F(HC3) df1 df2 p

,1506 ,0227 1473,2096 1,5728 3,0000 222,0000 ,1968

Model

coeff se(HC3) t p LLCI ULCI

constant 123,8016 43,7687 2,8285 ,0051 37,5462 210,0569

Kontroll 1,4050 5,3011 ,2650 ,7912 -9,0420 11,8519

ctq1ges -,0528 ,1972 -,2675 ,7893 -,4413 ,3358

mean_met -170,3848 93,2276 -1,8276 ,0689 -354,1093 13,3397

MSD does not predict mechanical pain threshold b=1.41, t(222)=.27, p=.79

Mean Methylation does not predict mechanical pain threshold b=-0.0004, t(222)=-2.71, p=0.01

Cumulative CTQ score does not predict mechanical pain threshold b=-.53, t(222)=-2.68, p=0.79

************************** TOTAL EFFECT MODEL ****************************

OUTCOME VARIABLE:

QST08T

Model Summary

R R-sq MSE F(HC3) df1 df2 p

,0009 ,0000 1493,9508 ,0002 1,0000 224,0000 ,9897

Model

coeff se(HC3) t p LLCI ULCI

constant 40,4408 3,2997 12,2560 ,0000 33,9384 46,9431

Kontroll -,0675 5,1979 -,0130 ,9897 -10,3104 10,1755

The total effect model is not significant F(1,224)=.0002, p=.99, R2=0.00 However, this is not a necessary requirement for mediation to occur. (Hayes ... Zhao, X., Lynch Jr, J. G., & Chen, Q. (2010). Reconsitering Baron and Kenny: Myths and truths about mediation analysis. Journal of consumer research, 37(2), 197-206.)

************** TOTAL, DIRECT, AND INDIRECT EFFECTS OF X ON Y **************

Total effect of X on Y

Effect se(HC3) t p LLCI ULCI c_ps

-,0675 5,1979 -,0130 ,9897 -10,3104 10,1755 -,0017

Direct effect of X on Y

Effect se(HC3) t p LLCI ULCI c'_ps

1,4050 5,3011 ,2650 ,7912 -9,0420 11,8519 ,0364

Indirect effect(s) of X on Y:

Effect BootSE BootLLCI BootULCI

TOTAL -1,4724 2,1786 -5,6174 3,1778

Ind1 -,5391 2,0040 -4,2108 3,7322

Ind2 -1,6203 1,1984 -4,4315 ,1062

Ind3 ,6870 ,5370 ,0097 2,0570

Cumulative CTQ Score and mean methylation mediated the effect of MSD on mechanical pain threshold, indirect effect=.69, SE=.54, 95% CI[0.01, 2.06]

Partially standardized indirect effect(s) of X on Y:

Effect BootSE BootLLCI BootULCI

TOTAL -,0382 ,0565 -,1472 ,0747

Ind1 -,0140 ,0516 -,1123 ,0881

Ind2 -,0420 ,0292 -,1082 ,0031

Ind3 ,0178 ,0124 ,0003 ,0478

Indirect effect key:

Ind1 Kontroll -> ctq1ges -> QST08T

Ind2 Kontroll -> mean_met -> QST08T

Ind3 Kontroll -> ctq1ges -> mean_met -> QST08T

*********************** ANALYSIS NOTES AND ERRORS ************************

Level of confidence for all confidence intervals in output:

95,0000

Number of bootstrap samples for percentile bootstrap confidence intervals:

10000

NOTE: A heteroscedasticity consistent standard error and covariance matrix estimator was used.

NOTE: Variables names longer than eight characters can produce incorrect output.

Shorter variable names are recommended.

------ END MATRIX -----

## Tactile control threshold control site

Run MATRIX procedure:

*************** PROCESS Procedure for SPSS Version 3.3 *******************

Written by Andrew F. Hayes, Ph.D. www.afhayes.com

Documentation available in Hayes (2018). www.guilford.com/p/hayes3

**************************************************************************

Model : 6

Y : QST07K tactile detection threshold control site

X : Kontroll

M1 : ctq1ges

M2 : mean_met

Sample

Size: 228

Custom

Seed: 1

**************************************************************************

OUTCOME VARIABLE:

ctq1ges

Model Summary

R R-sq MSE F(HC3) df1 df2 p

,3186 ,1015 225,2346 24,6753 1,0000 226,0000 ,0000

Model

coeff se(HC3) t p LLCI ULCI

constant 35,8654 1,0184 35,2182 ,0000 33,8587 37,8721

Kontroll 10,0468 2,0225 4,9674 ,0000 6,0613 14,0322

**************************************************************************

OUTCOME VARIABLE:

mean_met

Model Summary

R R-sq MSE F(HC3) df1 df2 p

,1845 ,0340 ,0012 4,1095 2,0000 225,0000 ,0177

Model

coeff se(HC3) t p LLCI ULCI

constant ,4920 ,0064 76,9144 ,0000 ,4794 ,5046

Kontroll ,0092 ,0049 1,8999 ,0587 -,0003 ,0188

ctq1ges -,0004 ,0001 -2,6704 ,0081 -,0007 -,0001

**************************************************************************

OUTCOME VARIABLE:

QST07K

Model Summary

R R-sq MSE F(HC3) df1 df2 p

,2923 ,0854 10,7788 4,5158 3,0000 224,0000 ,0043

Model

coeff se(HC3) t p LLCI ULCI

constant 14,3563 6,5936 2,1773 ,0305 1,3629 27,3498

Kontroll 1,3845 ,4277 3,2370 ,0014 ,5417 2,2274

ctq1ges -,0190 ,0171 -1,1098 ,2683 -,0527 ,0147

mean_met -24,4412 12,4713 -1,9598 ,0513 -49,0172 ,1349

Overall model of the influence of MSD, cumulative CTQ score and mean methylation on tactile detection threshold at the control site is significant F(3, 224)=4.52, p<0.01, R2=.09

MSD diagnoses does predict tactile detection threshold b=1.38, t(224)=3.24, p=0.001.

Cumulative CTQ score does not predict tactile detection threshold b=-0.02, t(224)=1.38, p=0.27.

Mean methylation does not predict tactile detection threshold b=-24.44, t(224)=-1.96, p=0.051.

************************** TOTAL EFFECT MODEL ****************************

OUTCOME VARIABLE:

QST07K

Model Summary

R R-sq MSE F(HC3) df1 df2 p

,1562 ,0244 11,3964 5,5490 1,0000 226,0000 ,0193

Model

coeff se(HC3) t p LLCI ULCI

constant 1,9885 ,2854 6,9668 ,0000 1,4260 2,5509

Kontroll 1,0633 ,4514 2,3556 ,0193 ,1738 1,9527

In the total effect model MSD diagnoses does predict tactile detection threshold b=1.06, t(226)=2.36, p=0.02.

************** TOTAL, DIRECT, AND INDIRECT EFFECTS OF X ON Y **************

Total effect of X on Y

Effect se(HC3) t p LLCI ULCI c_ps

1,0633 ,4514 2,3556 ,0193 ,1738 1,9527 ,3118

Direct effect of X on Y

Effect se(HC3) t p LLCI ULCI c'_ps

1,3845 ,4277 3,2370 ,0014 ,5417 2,2274 ,4060

Indirect effect(s) of X on Y:

Effect BootSE BootLLCI BootULCI

TOTAL -,3213 ,2509 -,8998 ,0906

Ind1 -,1909 ,1729 -,5528 ,1267

Ind2 -,2253 ,1731 -,6645 ,0044

Ind3 ,0949 ,0608 ,0116 ,2407

Partially standardized indirect effect(s) of X on Y:

Effect BootSE BootLLCI BootULCI

TOTAL -,0942 ,0650 -,2234 ,0295

Ind1 -,0560 ,0482 -,1494 ,0415

Ind2 -,0661 ,0428 -,1640 ,0013

Ind3 ,0278 ,0152 ,0041 ,0624

Cumulative CTQ Score and mean methylation mediated the effect of MSD on tactile detection threshold, indirect effect=.03, SE=.02, 95% CI[0.01, 0.06]

Indirect effect key:

Ind1 Kontroll -> ctq1ges -> QST07K

Ind2 Kontroll -> mean_met -> QST07K

Ind3 Kontroll -> ctq1ges -> mean_met -> QST07K

*********************** ANALYSIS NOTES AND ERRORS ************************

Level of confidence for all confidence intervals in output:

95,0000

Number of bootstrap samples for percentile bootstrap confidence intervals:

10000

NOTE: A heteroscedasticity consistent standard error and covariance matrix estimator was used.

NOTE: Variables names longer than eight characters can produce incorrect output.

Shorter variable names are recommended.

------ END MATRIX -----

## Tactile control threshold test site

Run MATRIX procedure:

*************** PROCESS Procedure for SPSS Version 3.3 *******************

Written by Andrew F. Hayes, Ph.D. www.afhayes.com

Documentation available in Hayes (2018). www.guilford.com/p/hayes3

**************************************************************************

Model : 6

Y : QST07T

X : Kontroll

M1 : ctq1ges

M2 : mean_met

Sample

Size: 228

Custom

Seed: 1

**************************************************************************

OUTCOME VARIABLE:

ctq1ges

Model Summary

R R-sq MSE F(HC3) df1 df2 p

,3186 ,1015 225,2346 24,6753 1,0000 226,0000 ,0000

Model

coeff se(HC3) t p LLCI ULCI

constant 35,8654 1,0184 35,2182 ,0000 33,8587 37,8721

Kontroll 10,0468 2,0225 4,9674 ,0000 6,0613 14,0322

**************************************************************************

OUTCOME VARIABLE:

mean_met

Model Summary

R R-sq MSE F(HC3) df1 df2 p

,1845 ,0340 ,0012 4,1095 2,0000 225,0000 ,0177

Model

coeff se(HC3) t p LLCI ULCI

constant ,4920 ,0064 76,9144 ,0000 ,4794 ,5046

Kontroll ,0092 ,0049 1,8999 ,0587 -,0003 ,0188

ctq1ges -,0004 ,0001 -2,6704 ,0081 -,0007 -,0001

**************************************************************************

OUTCOME VARIABLE:

QST07T

Model Summary

R R-sq MSE F(HC3) df1 df2 p

,2366 ,0560 186,9310 3,5352 3,0000 224,0000 ,0156

Model

coeff se(HC3) t p LLCI ULCI

constant 21,2949 12,4571 1,7095 ,0888 -3,2532 45,8430

Kontroll 6,0145 1,8925 3,1780 ,0017 2,2850 9,7439

ctq1ges ,0165 ,0756 ,2184 ,8273 -,1324 ,1654

mean_met -39,1371 23,5747 -1,6601 ,0983 -85,5936 7,3195

Overall model of the influence of MSD, cumulative CTQ score and mean methylation is significant F(3, 224)=3.54, p=0.02, R2=.06

MSD diagnoses does predict tactile detection threshold b=6.01, t(224)=3.18, p=0.01.

Cumulative CTQ score does not predict tactile detection threshold b=0.02, t(224)=.22, p=0.83.

Mean methylation does not predict tactile detection threshold b=-39.13, t(224)=-1.66, p=0.10.

************************** TOTAL EFFECT MODEL ****************************

OUTCOME VARIABLE:

QST07T

Model Summary

R R-sq MSE F(HC3) df1 df2 p

,2140 ,0458 187,2776 10,2203 1,0000 226,0000 ,0016

Model

coeff se(HC3) t p LLCI ULCI

constant 3,1735 ,2824 11,2372 ,0000 2,6170 3,7300

Kontroll 5,9715 1,8679 3,1969 ,0016 2,2908 9,6521

************** TOTAL, DIRECT, AND INDIRECT EFFECTS OF X ON Y **************

Total effect of X on Y

Effect se(HC3) t p LLCI ULCI c_ps

5,9715 1,8679 3,1969 ,0016 2,2908 9,6521 ,4272

In the total effect model MSD diagnoses does predict tactile detection threshold b=5.97, t(226)=3.20, p=0.002.

Direct effect of X on Y

Effect se(HC3) t p LLCI ULCI c'_ps

6,0145 1,8925 3,1780 ,0017 2,2850 9,7439 ,4303

Indirect effect(s) of X on Y:

Effect BootSE BootLLCI BootULCI

TOTAL -,0430 ,8308 -1,6104 1,7110

Ind1 ,1658 ,7591 -1,2081 1,8151

Ind2 -,3608 ,3136 -1,1574 ,0197

Ind3 ,1520 ,1169 ,0013 ,4470

Cumulative CTQ Score and mean methylation mediated the effect of MSD on tactile detection threshold, indirect effect=.15, SE=.12, 95% CI[0.001, 0.45]

Partially standardized indirect effect(s) of X on Y:

Effect BootSE BootLLCI BootULCI

TOTAL -,0031 ,0614 -,1360 ,1019

Ind1 ,0119 ,0539 -,1008 ,1080

Ind2 -,0258 ,0241 -,0879 ,0016

Ind3 ,0109 ,0090 ,0001 ,0345

Indirect effect key:

Ind1 Kontroll -> ctq1ges -> QST07T

Ind2 Kontroll -> mean_met -> QST07T

Ind3 Kontroll -> ctq1ges -> mean_met -> QST07T

*********************** ANALYSIS NOTES AND ERRORS ************************

Level of confidence for all confidence intervals in output:

95,0000

Number of bootstrap samples for percentile bootstrap confidence intervals:

10000

NOTE: A heteroscedasticity consistent standard error and covariance matrix estimator was used.

NOTE: Variables names longer than eight characters can produce incorrect output.

Shorter variable names are recommended.

------ END MATRIX -----

## Pressure pain threshold control site

Run MATRIX procedure:

*************** PROCESS Procedure for SPSS Version 3.3 *******************

Written by Andrew F. Hayes, Ph.D. www.afhayes.com

Documentation available in Hayes (2018). www.guilford.com/p/hayes3

**************************************************************************

Model : 6

Y : QST13K

X : Kontroll

M1 : ctq1ges

M2 : mean_met

Sample

Size: 227

Custom

Seed: 1

**************************************************************************

OUTCOME VARIABLE:

ctq1ges

Model Summary

R R-sq MSE F(HC3) df1 df2 p

,3164 ,1001 226,0244 24,2992 1,0000 225,0000 ,0000

Model

coeff se(HC3) t p LLCI ULCI

constant 35,9246 1,0255 35,0320 ,0000 33,9038 37,9453

Kontroll 9,9876 2,0261 4,9294 ,0000 5,9950 13,9802

**************************************************************************

OUTCOME VARIABLE:

mean_met

Model Summary

R R-sq MSE F(HC3) df1 df2 p

,1846 ,0341 ,0012 4,0990 2,0000 224,0000 ,0178

Model

coeff se(HC3) t p LLCI ULCI

constant ,4918 ,0064 76,4868 ,0000 ,4791 ,5045

Kontroll ,0093 ,0049 1,9206 ,0560 -,0002 ,0189

ctq1ges -,0004 ,0001 -2,6509 ,0086 -,0007 -,0001

**************************************************************************

OUTCOME VARIABLE:

QST13K

Model Summary

R R-sq MSE F(HC3) df1 df2 p

,5044 ,2544 10202,3661 24,3994 3,0000 223,0000 ,0000

Model

coeff se(HC3) t p LLCI ULCI

constant 425,9971 112,6778 3,7807 ,0002 203,9476 648,0467

Kontroll -113,5368 14,0480 -8,0821 ,0000 -141,2206 -85,8530

ctq1ges -,2310 ,5010 -,4610 ,6453 -1,2183 ,7564

mean_met -126,5058 223,4805 -,5661 ,5719 -566,9098 313,8981

************************** TOTAL EFFECT MODEL ****************************

OUTCOME VARIABLE:

QST13K

Model Summary

R R-sq MSE F(HC3) df1 df2 p

,5025 ,2525 10137,8058 74,4437 1,0000 225,0000 ,0000

Model

coeff se(HC3) t p LLCI ULCI

constant 357,2328 8,0832 44,1942 ,0000 341,3042 373,1613

Kontroll -116,5391 13,5070 -8,6281 ,0000 -143,1554 -89,9227

************** TOTAL, DIRECT, AND INDIRECT EFFECTS OF X ON Y **************

Total effect of X on Y

Effect se(HC3) t p LLCI ULCI c_ps

-116,5391 13,5070 -8,6281 ,0000 -143,1554 -89,9227 -1,0029

Direct effect of X on Y

Effect se(HC3) t p LLCI ULCI c'_ps

-113,5368 14,0480 -8,0821 ,0000 -141,2206 -85,8530 -,9771

Indirect effect(s) of X on Y:

Effect BootSE BootLLCI BootULCI

TOTAL -3,0023 5,3931 -13,5481 7,6228

Ind1 -2,3066 4,9852 -12,0362 7,6332

Ind2 -1,1814 2,3636 -6,8075 3,1630

Ind3 ,4857 ,9332 -1,3738 2,4839

Partially standardized indirect effect(s) of X on Y:

Effect BootSE BootLLCI BootULCI

TOTAL -,0258 ,0463 -,1141 ,0662

Ind1 -,0199 ,0429 -,1033 ,0662

Ind2 -,0102 ,0202 -,0573 ,0276

Ind3 ,0042 ,0080 -,0120 ,0210

Indirect effect key:

Ind1 Kontroll -> ctq1ges -> QST13K

Ind2 Kontroll -> mean_met -> QST13K

Ind3 Kontroll -> ctq1ges -> mean_met -> QST13K

*********************** ANALYSIS NOTES AND ERRORS ************************

Level of confidence for all confidence intervals in output:

95,0000

Number of bootstrap samples for percentile bootstrap confidence intervals:

10000

NOTE: A heteroscedasticity consistent standard error and covariance matrix estimator was used.

NOTE: Variables names longer than eight characters can produce incorrect output.

Shorter variable names are recommended.

------ END MATRIX -----

## Pressure pain threshold test site

Run MATRIX procedure:

*************** PROCESS Procedure for SPSS Version 3.3 *******************

Written by Andrew F. Hayes, Ph.D. www.afhayes.com

Documentation available in Hayes (2018). www.guilford.com/p/hayes3

**************************************************************************

Model : 6

Y : QST13T

X : Kontroll

M1 : ctq1ges

M2 : mean_met

Sample

Size: 227

Custom

Seed: 1

**************************************************************************

OUTCOME VARIABLE:

ctq1ges

Model Summary

R R-sq MSE F(HC3) df1 df2 p

,3164 ,1001 226,0244 24,2992 1,0000 225,0000 ,0000

Model

coeff se(HC3) t p LLCI ULCI

constant 35,9246 1,0255 35,0320 ,0000 33,9038 37,9453

Kontroll 9,9876 2,0261 4,9294 ,0000 5,9950 13,9802

**************************************************************************

OUTCOME VARIABLE:

mean_met

Model Summary

R R-sq MSE F(HC3) df1 df2 p

,1846 ,0341 ,0012 4,0990 2,0000 224,0000 ,0178

Model

coeff se(HC3) t p LLCI ULCI

constant ,4918 ,0064 76,4868 ,0000 ,4791 ,5045

Kontroll ,0093 ,0049 1,9206 ,0560 -,0002 ,0189

ctq1ges -,0004 ,0001 -2,6509 ,0086 -,0007 -,0001

**************************************************************************

OUTCOME VARIABLE:

QST13T

Model Summary

R R-sq MSE F(HC3) df1 df2 p

,5259 ,2766 16420,0863 30,0029 3,0000 223,0000 ,0000

Model

coeff se(HC3) t p LLCI ULCI

constant 438,8362 116,4843 3,7673 ,0002 209,2853 668,3871

Kontroll -156,5805 17,1173 -9,1475 ,0000 -190,3128 -122,8481

ctq1ges ,1244 ,6974 ,1784 ,8586 -1,2499 1,4987

mean_met -195,5848 231,1037 -,8463 ,3983 -651,0115 259,8419

************************** TOTAL EFFECT MODEL ****************************

OUTCOME VARIABLE:

QST13T

Model Summary

R R-sq MSE F(HC3) df1 df2 p

,5236 ,2742 16327,6265 83,3503 1,0000 225,0000 ,0000

Model

coeff se(HC3) t p LLCI ULCI

constant 349,8190 10,4176 33,5796 ,0000 329,2904 370,3475

Kontroll -156,4136 17,1325 -9,1296 ,0000 -190,1743 -122,6529

************** TOTAL, DIRECT, AND INDIRECT EFFECTS OF X ON Y **************

Total effect of X on Y

Effect se(HC3) t p LLCI ULCI c_ps

-156,4136 17,1325 -9,1296 ,0000 -190,1743 -122,6529 -1,0452

Direct effect of X on Y

Effect se(HC3) t p LLCI ULCI c'_ps

-156,5805 17,1173 -9,1475 ,0000 -190,3128 -122,8481 -1,0463

Indirect effect(s) of X on Y:

Effect BootSE BootLLCI BootULCI

TOTAL ,1669 7,2065 -12,8926 15,8941

Ind1 1,2424 6,8874 -11,2410 16,1379

Ind2 -1,8265 2,3570 -6,9683 2,6274

Ind3 ,7509 1,0014 -,9449 3,0925

Partially standardized indirect effect(s) of X on Y:

Effect BootSE BootLLCI BootULCI

TOTAL ,0011 ,0480 -,0877 ,1037

Ind1 ,0083 ,0458 -,0763 ,1055

Ind2 -,0122 ,0160 -,0473 ,0174

Ind3 ,0050 ,0068 -,0063 ,0208

Indirect effect key:

Ind1 Kontroll -> ctq1ges -> QST13T

Ind2 Kontroll -> mean_met -> QST13T

Ind3 Kontroll -> ctq1ges -> mean_met -> QST13T

*********************** ANALYSIS NOTES AND ERRORS ************************

Level of confidence for all confidence intervals in output:

95,0000

Number of bootstrap samples for percentile bootstrap confidence intervals:

10000

NOTE: A heteroscedasticity consistent standard error and covariance matrix estimator was used.

NOTE: Variables names longer than eight characters can produce incorrect output.

Shorter variable names are recommended.

------ END MATRIX -----

# Mean methylation of CpG -480/-429

## Mechanical pain threshold control site

Run MATRIX procedure:

*************** PROCESS Procedure for SPSS Version 3.3 *******************

Written by Andrew F. Hayes, Ph.D. www.afhayes.com

Documentation available in Hayes (2018). www.guilford.com/p/hayes3

**************************************************************************

Model : 6

Y : QST08K

X : Kontroll

M1 : ctq1ges

M2 : m2521_25

Sample

Size: 227

Custom

Seed: 1

**************************************************************************

OUTCOME VARIABLE:

ctq1ges

Model Summary

R R-sq MSE F(HC3) df1 df2 p

,3190 ,1017 226,1670 24,5207 1,0000 225,0000 ,0000

Model

coeff se(HC3) t p LLCI ULCI

constant 35,8654 1,0184 35,2182 ,0000 33,8586 37,8722

Kontroll 10,0823 2,0361 4,9518 ,0000 6,0701 14,0946

**************************************************************************

OUTCOME VARIABLE:

m2521_25

Model Summary

R R-sq MSE F(HC3) df1 df2 p

,2790 ,0778 ,0023 8,2662 2,0000 224,0000 ,0003

Model

coeff se(HC3) t p LLCI ULCI

constant ,3158 ,0094 33,4360 ,0000 ,2972 ,3344

Kontroll ,0100 ,0073 1,3805 ,1688 -,0043 ,0243

ctq1ges -,0009 ,0002 -4,0015 ,0001 -,0014 -,0005

**************************************************************************

OUTCOME VARIABLE:

QST08K

Model Summary

R R-sq MSE F(HC3) df1 df2 p

,1107 ,0122 2852,5789 1,0144 3,0000 223,0000 ,3871

Model

coeff se(HC3) t p LLCI ULCI

constant 71,7383 21,1594 3,3904 ,0008 30,0403 113,4363

Kontroll -11,7106 7,0680 -1,6568 ,0990 -25,6394 2,2181

ctq1ges ,0885 ,2421 ,3655 ,7151 -,3885 ,5655

m2521_25 -31,9007 52,9202 -,6028 ,5472 -136,1885 72,3870

************************** TOTAL EFFECT MODEL ****************************

OUTCOME VARIABLE:

QST08K

Model Summary

R R-sq MSE F(HC3) df1 df2 p

,1017 ,0104 2832,6560 2,3655 1,0000 225,0000 ,1254

Model

coeff se(HC3) t p LLCI ULCI

constant 65,8864 5,4508 12,0875 ,0000 55,1453 76,6275

Kontroll -10,8434 7,0502 -1,5380 ,1254 -24,7363 3,0495

************** TOTAL, DIRECT, AND INDIRECT EFFECTS OF X ON Y **************

Total effect of X on Y

Effect se(HC3) t p LLCI ULCI c_ps

-10,8434 7,0502 -1,5380 ,1254 -24,7363 3,0495 -,2031

Direct effect of X on Y

Effect se(HC3) t p LLCI ULCI c'_ps

-11,7106 7,0680 -1,6568 ,0990 -25,6394 2,2181 -,2194

Indirect effect(s) of X on Y:

Effect BootSE BootLLCI BootULCI

TOTAL ,8672 2,4767 -3,5392 6,3323

Ind1 ,8920 2,4403 -3,3907 6,3611

Ind2 -,3199 ,6680 -1,9516 ,7461

Ind3 ,2951 ,5115 -,6926 1,3670

Partially standardized indirect effect(s) of X on Y:

Effect BootSE BootLLCI BootULCI

TOTAL ,0162 ,0470 -,0649 ,1223

Ind1 ,0167 ,0464 -,0625 ,1226

Ind2 -,0060 ,0124 -,0360 ,0150

Ind3 ,0055 ,0096 -,0139 ,0246

Indirect effect key:

Ind1 Kontroll -> ctq1ges -> QST08K

Ind2 Kontroll -> m2521_25 -> QST08K

Ind3 Kontroll -> ctq1ges -> m2521_25 -> QST08K

*********************** ANALYSIS NOTES AND ERRORS ************************

Level of confidence for all confidence intervals in output:

95,0000

Number of bootstrap samples for percentile bootstrap confidence intervals:

10000

NOTE: A heteroscedasticity consistent standard error and covariance matrix estimator was used.

NOTE: Variables names longer than eight characters can produce incorrect output.

Shorter variable names are recommended.

------ END MATRIX -----

## Mechanical pain threshold test site

Run MATRIX procedure:

*************** PROCESS Procedure for SPSS Version 3.3 *******************

Written by Andrew F. Hayes, Ph.D. www.afhayes.com

Documentation available in Hayes (2018). www.guilford.com/p/hayes3

**************************************************************************

Model : 6

Y : QST08T

X : Kontroll

M1 : ctq1ges

M2 : m2521_25

Sample

Size: 226

Custom

Seed: 1

**************************************************************************

OUTCOME VARIABLE:

ctq1ges

Model Summary

R R-sq MSE F(HC3) df1 df2 p

,3228 ,1042 226,1701 24,9605 1,0000 224,0000 ,0000

Model

coeff se(HC3) t p LLCI ULCI

constant 35,8654 1,0184 35,2182 ,0000 33,8586 37,8722

Kontroll 10,2195 2,0455 4,9960 ,0000 6,1886 14,2504

**************************************************************************

OUTCOME VARIABLE:

m2521_25

Model Summary

R R-sq MSE F(HC3) df1 df2 p

,2825 ,0798 ,0023 8,3942 2,0000 223,0000 ,0003

Model

coeff se(HC3) t p LLCI ULCI

constant ,3163 ,0095 33,3587 ,0000 ,2976 ,3350

Kontroll ,0105 ,0073 1,4413 ,1509 -,0039 ,0250

ctq1ges -,0009 ,0002 -4,0370 ,0001 -,0014 -,0005

**************************************************************************

OUTCOME VARIABLE:

QST08T

Model Summary

R R-sq MSE F(HC3) df1 df2 p

,0653 ,0043 1500,9789 ,3754 3,0000 222,0000 ,7708

Model

coeff se(HC3) t p LLCI ULCI

constant 56,6551 17,4165 3,2530 ,0013 22,3322 90,9779

Kontroll ,3426 5,1582 ,0664 ,9471 -9,8227 10,5079

ctq1ges -,0347 ,2023 -,1718 ,8637 -,4333 ,3638

m2521_25 -52,9081 56,0270 -,9443 ,3460 -163,3210 57,5048

************************** TOTAL EFFECT MODEL ****************************

OUTCOME VARIABLE:

QST08T

Model Summary

R R-sq MSE F(HC3) df1 df2 p

,0009 ,0000 1493,9508 ,0002 1,0000 224,0000 ,9897

Model

coeff se(HC3) t p LLCI ULCI

constant 40,4408 3,2997 12,2560 ,0000 33,9384 46,9431

Kontroll -,0675 5,1979 -,0130 ,9897 -10,3104 10,1755

************** TOTAL, DIRECT, AND INDIRECT EFFECTS OF X ON Y **************

Total effect of X on Y

Effect se(HC3) t p LLCI ULCI c_ps

-,0675 5,1979 -,0130 ,9897 -10,3104 10,1755 -,0017

Direct effect of X on Y

Effect se(HC3) t p LLCI ULCI c'_ps

,3426 5,1582 ,0664 ,9471 -9,8227 10,5079 ,0089

Indirect effect(s) of X on Y:

Effect BootSE BootLLCI BootULCI

TOTAL -,4101 2,1627 -4,3498 4,3009

Ind1 -,3551 2,0777 -4,1150 4,1571

Ind2 -,5580 ,8072 -2,6607 ,5431

Ind3 ,5030 ,5840 -,4726 1,8804

Partially standardized indirect effect(s) of X on Y:

Effect BootSE BootLLCI BootULCI

TOTAL -,0106 ,0553 -,1148 ,1008

Ind1 -,0092 ,0532 -,1085 ,0987

Ind2 -,0145 ,0202 -,0641 ,0157

Ind3 ,0130 ,0143 -,0138 ,0436

Indirect effect key:

Ind1 Kontroll -> ctq1ges -> QST08T

Ind2 Kontroll -> m2521_25 -> QST08T

Ind3 Kontroll -> ctq1ges -> m2521_25 -> QST08T

*********************** ANALYSIS NOTES AND ERRORS ************************

Level of confidence for all confidence intervals in output:

95,0000

Number of bootstrap samples for percentile bootstrap confidence intervals:

10000

NOTE: A heteroscedasticity consistent standard error and covariance matrix estimator was used.

NOTE: Variables names longer than eight characters can produce incorrect output.

Shorter variable names are recommended.

------ END MATRIX -----

## Tactile control threshold control site

Run MATRIX procedure:

*************** PROCESS Procedure for SPSS Version 3.3 *******************

Written by Andrew F. Hayes, Ph.D. www.afhayes.com

Documentation available in Hayes (2018). www.guilford.com/p/hayes3

**************************************************************************

Model : 6

Y : QST07K

X : Kontroll

M1 : ctq1ges

M2 : m2521_25

Sample

Size: 228

Custom

Seed: 1

**************************************************************************

OUTCOME VARIABLE:

ctq1ges

Model Summary

R R-sq MSE F(HC3) df1 df2 p

,3186 ,1015 225,2346 24,6753 1,0000 226,0000 ,0000

Model

coeff se(HC3) t p LLCI ULCI

constant 35,8654 1,0184 35,2182 ,0000 33,8587 37,8721

Kontroll 10,0468 2,0225 4,9674 ,0000 6,0613 14,0322

**************************************************************************

OUTCOME VARIABLE:

m2521_25

Model Summary

R R-sq MSE F(HC3) df1 df2 p

,2790 ,0778 ,0023 8,2690 2,0000 225,0000 ,0003

Model

coeff se(HC3) t p LLCI ULCI

constant ,3158 ,0094 33,4596 ,0000 ,2972 ,3344

Kontroll ,0100 ,0072 1,3863 ,1670 -,0042 ,0242

ctq1ges -,0009 ,0002 -4,0045 ,0001 -,0014 -,0005

**************************************************************************

OUTCOME VARIABLE:

QST07K

Model Summary

R R-sq MSE F(HC3) df1 df2 p

,1848 ,0341 11,3832 2,6869 3,0000 224,0000 ,0473

Model

coeff se(HC3) t p LLCI ULCI

constant 4,3668 2,2253 1,9623 ,0510 -,0185 8,7521

Kontroll 1,2237 ,4428 2,7638 ,0062 ,3512 2,0962

ctq1ges -,0155 ,0183 -,8469 ,3979 -,0514 ,0205

m2521_25 -6,4464 5,5760 -1,1561 ,2489 -17,4346 4,5417

************************** TOTAL EFFECT MODEL ****************************

OUTCOME VARIABLE:

QST07K

Model Summary

R R-sq MSE F(HC3) df1 df2 p

,1562 ,0244 11,3964 5,5490 1,0000 226,0000 ,0193

Model

coeff se(HC3) t p LLCI ULCI

constant 1,9885 ,2854 6,9668 ,0000 1,4260 2,5509

Kontroll 1,0633 ,4514 2,3556 ,0193 ,1738 1,9527

************** TOTAL, DIRECT, AND INDIRECT EFFECTS OF X ON Y **************

Total effect of X on Y

Effect se(HC3) t p LLCI ULCI c_ps

1,0633 ,4514 2,3556 ,0193 ,1738 1,9527 ,3118

Direct effect of X on Y

Effect se(HC3) t p LLCI ULCI c'_ps

1,2237 ,4428 2,7638 ,0062 ,3512 2,0962 ,3588

Indirect effect(s) of X on Y:

Effect BootSE BootLLCI BootULCI

TOTAL -,1604 ,1826 -,5195 ,2102

Ind1 -,1554 ,1824 -,5179 ,2043

Ind2 -,0645 ,0770 -,2402 ,0649

Ind3 ,0594 ,0551 -,0433 ,1785

Partially standardized indirect effect(s) of X on Y:

Effect BootSE BootLLCI BootULCI

TOTAL -,0470 ,0535 -,1477 ,0657

Ind1 -,0456 ,0532 -,1477 ,0635

Ind2 -,0189 ,0234 -,0730 ,0192

Ind3 ,0174 ,0166 -,0127 ,0539

Indirect effect key:

Ind1 Kontroll -> ctq1ges -> QST07K

Ind2 Kontroll -> m2521_25 -> QST07K

Ind3 Kontroll -> ctq1ges -> m2521_25 -> QST07K

*********************** ANALYSIS NOTES AND ERRORS ************************

Level of confidence for all confidence intervals in output:

95,0000

Number of bootstrap samples for percentile bootstrap confidence intervals:

10000

NOTE: A heteroscedasticity consistent standard error and covariance matrix estimator was used.

NOTE: Variables names longer than eight characters can produce incorrect output.

Shorter variable names are recommended.

------ END MATRIX -----

## Tactile control threshold test site

Run MATRIX procedure:

*************** PROCESS Procedure for SPSS Version 3.3 *******************

Written by Andrew F. Hayes, Ph.D. www.afhayes.com

Documentation available in Hayes (2018). www.guilford.com/p/hayes3

**************************************************************************

Model : 6

Y : QST07T

X : Kontroll

M1 : ctq1ges

M2 : m2521_25

Sample

Size: 228

Custom

Seed: 1

**************************************************************************

OUTCOME VARIABLE:

ctq1ges

Model Summary

R R-sq MSE F(HC3) df1 df2 p

,3186 ,1015 225,2346 24,6753 1,0000 226,0000 ,0000

Model

coeff se(HC3) t p LLCI ULCI

constant 35,8654 1,0184 35,2182 ,0000 33,8587 37,8721

Kontroll 10,0468 2,0225 4,9674 ,0000 6,0613 14,0322

**************************************************************************

OUTCOME VARIABLE:

m2521_25

Model Summary

R R-sq MSE F(HC3) df1 df2 p

,2790 ,0778 ,0023 8,2690 2,0000 225,0000 ,0003

Model

coeff se(HC3) t p LLCI ULCI

constant ,3158 ,0094 33,4596 ,0000 ,2972 ,3344

Kontroll ,0100 ,0072 1,3863 ,1670 -,0042 ,0242

ctq1ges -,0009 ,0002 -4,0045 ,0001 -,0014 -,0005

**************************************************************************

OUTCOME VARIABLE:

QST07T

Model Summary

R R-sq MSE F(HC3) df1 df2 p

,2229 ,0497 188,1780 4,1457 3,0000 224,0000 ,0069

Model

coeff se(HC3) t p LLCI ULCI

constant -2,8535 11,9755 -,2383 ,8119 -26,4526 20,7456

Kontroll 5,4986 1,7342 3,1708 ,0017 2,0813 8,9160

ctq1ges ,0458 ,0969 ,4730 ,6367 -,1452 ,2369

m2521_25 15,4916 31,3113 ,4948 ,6213 -46,2108 77,1940

************************** TOTAL EFFECT MODEL ****************************

OUTCOME VARIABLE:

QST07T

Model Summary

R R-sq MSE F(HC3) df1 df2 p

,2140 ,0458 187,2776 10,2203 1,0000 226,0000 ,0016

Model

coeff se(HC3) t p LLCI ULCI

constant 3,1735 ,2824 11,2372 ,0000 2,6170 3,7300

Kontroll 5,9715 1,8679 3,1969 ,0016 2,2908 9,6521

************** TOTAL, DIRECT, AND INDIRECT EFFECTS OF X ON Y **************

Total effect of X on Y

Effect se(HC3) t p LLCI ULCI c_ps

5,9715 1,8679 3,1969 ,0016 2,2908 9,6521 ,4272

Direct effect of X on Y

Effect se(HC3) t p LLCI ULCI c'_ps

5,4986 1,7342 3,1708 ,0017 2,0813 8,9160 ,3934

Indirect effect(s) of X on Y:

Effect BootSE BootLLCI BootULCI

TOTAL ,4728 1,0081 -1,1528 2,8331

Ind1 ,4606 ,9602 -1,1208 2,6205

Ind2 ,1550 ,3729 -,4374 1,1108

Ind3 -,1428 ,2866 -,8058 ,3431

Partially standardized indirect effect(s) of X on Y:

Effect BootSE BootLLCI BootULCI

TOTAL ,0338 ,0663 -,0991 ,1586

Ind1 ,0330 ,0640 -,0965 ,1491

Ind2 ,0111 ,0242 -,0416 ,0602

Ind3 -,0102 ,0193 -,0440 ,0325

Indirect effect key:

Ind1 Kontroll -> ctq1ges -> QST07T

Ind2 Kontroll -> m2521_25 -> QST07T

Ind3 Kontroll -> ctq1ges -> m2521_25 -> QST07T

*********************** ANALYSIS NOTES AND ERRORS ************************

Level of confidence for all confidence intervals in output:

95,0000

Number of bootstrap samples for percentile bootstrap confidence intervals:

10000

NOTE: A heteroscedasticity consistent standard error and covariance matrix estimator was used.

NOTE: Variables names longer than eight characters can produce incorrect output.

Shorter variable names are recommended.

------ END MATRIX -----

## Pressure pain threshold control site

Run MATRIX procedure:

*************** PROCESS Procedure for SPSS Version 3.3 *******************

Written by Andrew F. Hayes, Ph.D. www.afhayes.com

Documentation available in Hayes (2018). www.guilford.com/p/hayes3

**************************************************************************

Model : 6

Y : QST13T

X : Kontroll

M1 : ctq1ges

M2 : m2521_25

Sample

Size: 227

Custom

Seed: 1

**************************************************************************

OUTCOME VARIABLE:

ctq1ges

Model Summary

R R-sq MSE F(HC3) df1 df2 p

,3164 ,1001 226,0244 24,2992 1,0000 225,0000 ,0000

Model

coeff se(HC3) t p LLCI ULCI

constant 35,9246 1,0255 35,0320 ,0000 33,9038 37,9453

Kontroll 9,9876 2,0261 4,9294 ,0000 5,9950 13,9802

**************************************************************************

OUTCOME VARIABLE:

m2521_25

Model Summary

R R-sq MSE F(HC3) df1 df2 p

,2781 ,0774 ,0023 8,1506 2,0000 224,0000 ,0004

Model

coeff se(HC3) t p LLCI ULCI

constant ,3155 ,0095 33,2851 ,0000 ,2968 ,3341

Kontroll ,0102 ,0072 1,4109 ,1596 -,0040 ,0244

ctq1ges -,0009 ,0002 -3,9842 ,0001 -,0014 -,0005

The overall model when performing regression on average methylation at CpGs -480 and -429 with CTQ score and MSD Diagnosis as predictors is significant F(2, 224)=8.15, p=0.0004, R2=.08

MSD diagnoses does not predict mean methylation b=0.01, t(224)=1.41, p=0.16

Cumulative CTQ score predicts mean methylation b=-0.0009, t(224)=-3.9, p=0.001

**************************************************************************

OUTCOME VARIABLE:

QST13T

Model Summary

R R-sq MSE F(HC3) df1 df2 p

,5324 ,2835 16263,0732 35,7094 3,0000 223,0000 ,0000

Model

coeff se(HC3) t p LLCI ULCI

constant 436,6232 54,4075 8,0251 ,0000 329,4046 543,8419

Kontroll -155,3706 17,3439 -8,9582 ,0000 -189,5495 -121,1917

ctq1ges -,0727 ,6823 -,1065 ,9153 -1,4173 1,2720

m2521_25 -297,8931 165,4498 -1,8005 ,0731 -623,9384 28,1522

Overall model of the influence of MSD, cumulative CTQ score and average methylation at CpGs -480 and -429 is not significant F(3, 223)=35.71, p<0.0001, R2=.28

MSD diagnoses does predict pressure pain threshold b=-155.37, t(223)=-8.96, p<0.001.

Cumulative CTQ score does not predict pressure pain threshold b=-0.07, t(223)=-0.10, p=0.91.

Average methylation at CpGs -480 and -429 does not predict pressure pain threshold b=-297,89 t(223)=165.45, p=0.073.

************************** TOTAL EFFECT MODEL ****************************

OUTCOME VARIABLE:

QST13T

Model Summary

R R-sq MSE F(HC3) df1 df2 p

,5236 ,2742 16327,6265 83,3503 1,0000 225,0000 ,0000

Model

coeff se(HC3) t p LLCI ULCI

constant 349,8190 10,4176 33,5796 ,0000 329,2904 370,3475

Kontroll -156,4136 17,1325 -9,1296 ,0000 -190,1743 -122,6529

************** TOTAL, DIRECT, AND INDIRECT EFFECTS OF X ON Y **************

Total effect of X on Y

Effect se(HC3) t p LLCI ULCI c_ps

-156,4136 17,1325 -9,1296 ,0000 -190,1743 -122,6529 -1,0452

In the total effect model MSD diagnoses does predict pressure pain threshold b=-156.41, t(225)=-9.13, p<0.001.

Direct effect of X on Y

Effect se(HC3) t p LLCI ULCI c'_ps

-155,3706 17,3439 -8,9582 ,0000 -189,5495 -121,1917 -1,0382

Indirect effect(s) of X on Y:

Effect BootSE BootLLCI BootULCI

TOTAL -1,0430 7,1507 -13,9923 14,6388

Ind1 -,7259 6,6820 -13,0896 13,6332

Ind2 -3,0363 2,6172 -9,0090 1,3353

Ind3 2,7192 1,6037 ,0148 6,2802

Partially standardized indirect effect(s) of X on Y:

Effect BootSE BootLLCI BootULCI

TOTAL -,0070 ,0478 -,0945 ,0957

Ind1 -,0049 ,0446 -,0886 ,0890

Ind2 -,0203 ,0173 -,0596 ,0089

Ind3 ,0182 ,0105 ,0001 ,0412

Cumulative CTQ Score and Average methylation at CpGs -480 and -429 mediated the effect of MSD pressure pain threshold, indirect effect=2.72, SE=1.60, 95% CI[0.015, 6.28]

Indirect effect key:

Ind1 Kontroll -> ctq1ges -> QST13T

Ind2 Kontroll -> m2521_25 -> QST13T

Ind3 Kontroll -> ctq1ges -> m2521_25 -> QST13T

*********************** ANALYSIS NOTES AND ERRORS ************************

Level of confidence for all confidence intervals in output:

95,0000

Number of bootstrap samples for percentile bootstrap confidence intervals:

10000

NOTE: A heteroscedasticity consistent standard error and covariance matrix estimator was used.

NOTE: Variables names longer than eight characters can produce incorrect output.

Shorter variable names are recommended.

------ END MATRIX -----

## Pressure pain threshold test site

Run MATRIX procedure:

*************** PROCESS Procedure for SPSS Version 3.3 *******************

Written by Andrew F. Hayes, Ph.D. www.afhayes.com

Documentation available in Hayes (2018). www.guilford.com/p/hayes3

**************************************************************************

Model : 6

Y : QST13T

X : Kontroll

M1 : ctq1ges

M2 : m2521_25

Sample

Size: 227

Custom

Seed: 1

**************************************************************************

OUTCOME VARIABLE:

ctq1ges

Model Summary

R R-sq MSE F(HC3) df1 df2 p

,3164 ,1001 226,0244 24,2992 1,0000 225,0000 ,0000

Model

coeff se(HC3) t p LLCI ULCI

constant 35,9246 1,0255 35,0320 ,0000 33,9038 37,9453

Kontroll 9,9876 2,0261 4,9294 ,0000 5,9950 13,9802

**************************************************************************

OUTCOME VARIABLE:

m2521_25

Model Summary

R R-sq MSE F(HC3) df1 df2 p

,2781 ,0774 ,0023 8,1506 2,0000 224,0000 ,0004

Model

coeff se(HC3) t p LLCI ULCI

constant ,3155 ,0095 33,2851 ,0000 ,2968 ,3341

Kontroll ,0102 ,0072 1,4109 ,1596 -,0040 ,0244

ctq1ges -,0009 ,0002 -3,9842 ,0001 -,0014 -,0005

**************************************************************************

OUTCOME VARIABLE:

QST13T

Model Summary

R R-sq MSE F(HC3) df1 df2 p

,5324 ,2835 16263,0732 35,7094 3,0000 223,0000 ,0000

Model

coeff se(HC3) t p LLCI ULCI

constant 436,6232 54,4075 8,0251 ,0000 329,4046 543,8419

Kontroll -155,3706 17,3439 -8,9582 ,0000 -189,5495 -121,1917

ctq1ges -,0727 ,6823 -,1065 ,9153 -1,4173 1,2720

m2521_25 -297,8931 165,4498 -1,8005 ,0731 -623,9384 28,1522

************************** TOTAL EFFECT MODEL ****************************

OUTCOME VARIABLE:

QST13T

Model Summary

R R-sq MSE F(HC3) df1 df2 p

,5236 ,2742 16327,6265 83,3503 1,0000 225,0000 ,0000

Model

coeff se(HC3) t p LLCI ULCI

constant 349,8190 10,4176 33,5796 ,0000 329,2904 370,3475

Kontroll -156,4136 17,1325 -9,1296 ,0000 -190,1743 -122,6529

************** TOTAL, DIRECT, AND INDIRECT EFFECTS OF X ON Y **************

Total effect of X on Y

Effect se(HC3) t p LLCI ULCI c_ps

-156,4136 17,1325 -9,1296 ,0000 -190,1743 -122,6529 -1,0452

Direct effect of X on Y

Effect se(HC3) t p LLCI ULCI c'_ps

-155,3706 17,3439 -8,9582 ,0000 -189,5495 -121,1917 -1,0382

Indirect effect(s) of X on Y:

Effect BootSE BootLLCI BootULCI

TOTAL -1,0430 7,1507 -13,9923 14,6388

Ind1 -,7259 6,6820 -13,0896 13,6332

Ind2 -3,0363 2,6172 -9,0090 1,3353

Ind3 2,7192 1,6037 ,0148 6,2802

Partially standardized indirect effect(s) of X on Y:

Effect BootSE BootLLCI BootULCI

TOTAL -,0070 ,0478 -,0945 ,0957

Ind1 -,0049 ,0446 -,0886 ,0890

Ind2 -,0203 ,0173 -,0596 ,0089

Ind3 ,0182 ,0105 ,0001 ,0412

Indirect effect key:

Ind1 Kontroll -> ctq1ges -> QST13T

Ind2 Kontroll -> m2521_25 -> QST13T

Ind3 Kontroll -> ctq1ges -> m2521_25 -> QST13T

*********************** ANALYSIS NOTES AND ERRORS ************************

Level of confidence for all confidence intervals in output:

95,0000

Number of bootstrap samples for percentile bootstrap confidence intervals:

10000

NOTE: A heteroscedasticity consistent standard error and covariance matrix estimator was used.

NOTE: Variables names longer than eight characters can produce incorrect output.

Shorter variable names are recommended.

------ END MATRIX -----

# CpG -628 (Bell / Gombert)

## Mechanical pain threshold control site

Run MATRIX procedure:

*************** PROCESS Procedure for SPSS Version 3.3 *******************

Written by Andrew F. Hayes, Ph.D. www.afhayes.com

Documentation available in Hayes (2018). www.guilford.com/p/hayes3

**************************************************************************

Model : 6

Y : QST08K

X : Kontroll

M1 : ctq1ges

M2 : m2373

Sample

Size: 227

Custom

Seed: 1

**************************************************************************

OUTCOME VARIABLE:

ctq1ges

Model Summary

R R-sq MSE F(HC3) df1 df2 p

,3190 ,1017 226,1670 24,5207 1,0000 225,0000 ,0000

Model

coeff se(HC3) t p LLCI ULCI

constant 35,8654 1,0184 35,2182 ,0000 33,8586 37,8722

Kontroll 10,0823 2,0361 4,9518 ,0000 6,0701 14,0946

**************************************************************************

OUTCOME VARIABLE:

m2373

Model Summary

R R-sq MSE F(HC3) df1 df2 p

,1690 ,0286 ,0039 3,0816 2,0000 224,0000 ,0478

Model

coeff se(HC3) t p LLCI ULCI

constant ,5562 ,0123 45,3631 ,0000 ,5321 ,5804

Kontroll ,0196 ,0088 2,2284 ,0268 ,0023 ,0370

ctq1ges -,0005 ,0003 -1,7293 ,0851 -,0011 ,0001

**************************************************************************

OUTCOME VARIABLE:

QST08K

Model Summary

R R-sq MSE F(HC3) df1 df2 p

,1086 ,0118 2853,8825 1,0790 3,0000 223,0000 ,3588

Model

coeff se(HC3) t p LLCI ULCI

constant 70,6010 40,2697 1,7532 ,0809 -8,7569 149,9589

Kontroll -11,7155 7,6486 -1,5317 ,1270 -26,7882 3,3573

ctq1ges ,1093 ,2353 ,4644 ,6428 -,3544 ,5729

m2373 -16,0683 73,7039 -,2180 ,8276 -161,3137 129,1770

************************** TOTAL EFFECT MODEL ****************************

OUTCOME VARIABLE:

QST08K

Model Summary

R R-sq MSE F(HC3) df1 df2 p

,1017 ,0104 2832,6560 2,3655 1,0000 225,0000 ,1254

Model

coeff se(HC3) t p LLCI ULCI

constant 65,8864 5,4508 12,0875 ,0000 55,1453 76,6275

Kontroll -10,8434 7,0502 -1,5380 ,1254 -24,7363 3,0495

************** TOTAL, DIRECT, AND INDIRECT EFFECTS OF X ON Y **************

Total effect of X on Y

Effect se(HC3) t p LLCI ULCI c_ps

-10,8434 7,0502 -1,5380 ,1254 -24,7363 3,0495 -,2031

Direct effect of X on Y

Effect se(HC3) t p LLCI ULCI c'_ps

-11,7155 7,6486 -1,5317 ,1270 -26,7882 3,3573 -,2195

Indirect effect(s) of X on Y:

Effect BootSE BootLLCI BootULCI

TOTAL ,8720 2,7121 -4,2109 6,5349

Ind1 1,1015 2,3750 -3,0833 6,2832

Ind2 -,3151 1,5039 -3,7267 2,4692

Ind3 ,0856 ,4582 -,7553 1,1619

Partially standardized indirect effect(s) of X on Y:

Effect BootSE BootLLCI BootULCI

TOTAL ,0163 ,0513 -,0820 ,1207

Ind1 ,0206 ,0450 -,0585 ,1198

Ind2 -,0059 ,0286 -,0725 ,0449

Ind3 ,0016 ,0087 -,0135 ,0230

Indirect effect key:

Ind1 Kontroll -> ctq1ges -> QST08K

Ind2 Kontroll -> m2373 -> QST08K

Ind3 Kontroll -> ctq1ges -> m2373 -> QST08K

*********************** ANALYSIS NOTES AND ERRORS ************************

Level of confidence for all confidence intervals in output:

95,0000

Number of bootstrap samples for percentile bootstrap confidence intervals:

10000

NOTE: A heteroscedasticity consistent standard error and covariance matrix estimator was used.

NOTE: Variables names longer than eight characters can produce incorrect output.

Shorter variable names are recommended.

------ END MATRIX -----

## Mechanical pain threshold test site

Run MATRIX procedure:

*************** PROCESS Procedure for SPSS Version 3.3 *******************

Written by Andrew F. Hayes, Ph.D. www.afhayes.com

Documentation available in Hayes (2018). www.guilford.com/p/hayes3

**************************************************************************

Model : 6

Y : QST08T

X : Kontroll

M1 : ctq1ges

M2 : m2373

Sample

Size: 226

Custom

Seed: 1

**************************************************************************

OUTCOME VARIABLE:

ctq1ges

Model Summary

R R-sq MSE F(HC3) df1 df2 p

,3228 ,1042 226,1701 24,9605 1,0000 224,0000 ,0000

Model

coeff se(HC3) t p LLCI ULCI

constant 35,8654 1,0184 35,2182 ,0000 33,8586 37,8722

Kontroll 10,2195 2,0455 4,9960 ,0000 6,1886 14,2504

**************************************************************************

OUTCOME VARIABLE:

m2373

Model Summary

R R-sq MSE F(HC3) df1 df2 p

,1756 ,0308 ,0039 3,3240 2,0000 223,0000 ,0378

Model

coeff se(HC3) t p LLCI ULCI

constant ,5570 ,0123 45,3655 ,0000 ,5328 ,5812

Kontroll ,0205 ,0088 2,3187 ,0213 ,0031 ,0378

ctq1ges -,0005 ,0003 -1,7940 ,0742 -,0012 ,0001

**************************************************************************

OUTCOME VARIABLE:

QST08T

Model Summary

R R-sq MSE F(HC3) df1 df2 p

,0663 ,0044 1500,7861 ,3428 3,0000 222,0000 ,7944

Model

coeff se(HC3) t p LLCI ULCI

constant 62,7152 30,2483 2,0733 ,0393 3,1046 122,3257

Kontroll ,6219 5,5293 ,1125 ,9106 -10,2747 11,5185

ctq1ges -,0080 ,2210 -,0362 ,9711 -,4435 ,4275

m2373 -40,9243 50,6892 -,8074 ,4203 -140,8179 58,9692

************************** TOTAL EFFECT MODEL ****************************

OUTCOME VARIABLE:

QST08T

Model Summary

R R-sq MSE F(HC3) df1 df2 p

,0009 ,0000 1493,9508 ,0002 1,0000 224,0000 ,9897

Model

coeff se(HC3) t p LLCI ULCI

constant 40,4408 3,2997 12,2560 ,0000 33,9384 46,9431

Kontroll -,0675 5,1979 -,0130 ,9897 -10,3104 10,1755

************** TOTAL, DIRECT, AND INDIRECT EFFECTS OF X ON Y **************

Total effect of X on Y

Effect se(HC3) t p LLCI ULCI c_ps

-,0675 5,1979 -,0130 ,9897 -10,3104 10,1755 -,0017

Direct effect of X on Y

Effect se(HC3) t p LLCI ULCI c'_ps

,6219 5,5293 ,1125 ,9106 -10,2747 11,5185 ,0161

Indirect effect(s) of X on Y:

Effect BootSE BootLLCI BootULCI

TOTAL -,6894 2,6545 -5,5091 5,0205

Ind1 -,0818 2,2764 -3,9662 5,0280

Ind2 -,8372 1,1658 -3,5553 1,1013

Ind3 ,2297 ,3578 -,3573 1,0814

Partially standardized indirect effect(s) of X on Y:

Effect BootSE BootLLCI BootULCI

TOTAL -,0179 ,0688 -,1522 ,1174

Ind1 -,0021 ,0579 -,1083 ,1164

Ind2 -,0217 ,0313 -,0958 ,0278

Ind3 ,0060 ,0096 -,0089 ,0296

Indirect effect key:

Ind1 Kontroll -> ctq1ges -> QST08T

Ind2 Kontroll -> m2373 -> QST08T

Ind3 Kontroll -> ctq1ges -> m2373 -> QST08T

*********************** ANALYSIS NOTES AND ERRORS ************************

Level of confidence for all confidence intervals in output:

95,0000

Number of bootstrap samples for percentile bootstrap confidence intervals:

10000

NOTE: A heteroscedasticity consistent standard error and covariance matrix estimator was used.

NOTE: Variables names longer than eight characters can produce incorrect output.

Shorter variable names are recommended.

------ END MATRIX -----

## Tactile control threshold control site

Run MATRIX procedure:

*************** PROCESS Procedure for SPSS Version 3.3 *******************

Written by Andrew F. Hayes, Ph.D. www.afhayes.com

Documentation available in Hayes (2018). www.guilford.com/p/hayes3

**************************************************************************

Model : 6

Y : QST07K

X : Kontroll

M1 : ctq1ges

M2 : m2373

Sample

Size: 228

Custom

Seed: 1

**************************************************************************

OUTCOME VARIABLE:

ctq1ges

Model Summary

R R-sq MSE F(HC3) df1 df2 p

,3186 ,1015 225,2346 24,6753 1,0000 226,0000 ,0000

Model

coeff se(HC3) t p LLCI ULCI

constant 35,8654 1,0184 35,2182 ,0000 33,8587 37,8721

Kontroll 10,0468 2,0225 4,9674 ,0000 6,0613 14,0322

**************************************************************************

OUTCOME VARIABLE:

m2373

Model Summary

R R-sq MSE F(HC3) df1 df2 p

,1665 ,0277 ,0039 2,9940 2,0000 225,0000 ,0521

Model

coeff se(HC3) t p LLCI ULCI

constant ,5561 ,0123 45,3600 ,0000 ,5319 ,5803

Kontroll ,0192 ,0088 2,1879 ,0297 ,0019 ,0365

ctq1ges -,0005 ,0003 -1,7187 ,0870 -,0011 ,0001

**************************************************************************

OUTCOME VARIABLE:

QST07K

Model Summary

R R-sq MSE F(HC3) df1 df2 p

,2385 ,0569 11,1151 3,8350 3,0000 224,0000 ,0105

Model

coeff se(HC3) t p LLCI ULCI

constant 7,6763 2,4677 3,1107 ,0021 2,8133 12,5392

Kontroll 1,3436 ,4510 2,9791 ,0032 ,4548 2,2323

ctq1ges -,0146 ,0147 -,9952 ,3207 -,0435 ,0143

m2373 -9,6121 3,8340 -2,5071 ,0129 -17,1674 -2,0569

************************** TOTAL EFFECT MODEL ****************************

OUTCOME VARIABLE:

QST07K

Model Summary

R R-sq MSE F(HC3) df1 df2 p

,1562 ,0244 11,3964 5,5490 1,0000 226,0000 ,0193

Model

coeff se(HC3) t p LLCI ULCI

constant 1,9885 ,2854 6,9668 ,0000 1,4260 2,5509

Kontroll 1,0633 ,4514 2,3556 ,0193 ,1738 1,9527

************** TOTAL, DIRECT, AND INDIRECT EFFECTS OF X ON Y **************

Total effect of X on Y

Effect se(HC3) t p LLCI ULCI c_ps

1,0633 ,4514 2,3556 ,0193 ,1738 1,9527 ,3118

Direct effect of X on Y

Effect se(HC3) t p LLCI ULCI c'_ps

1,3436 ,4510 2,9791 ,0032 ,4548 2,2323 ,3940

Indirect effect(s) of X on Y:

Effect BootSE BootLLCI BootULCI

TOTAL -,2803 ,1892 -,6806 ,0648

Ind1 -,1467 ,1493 -,4566 ,1362

Ind2 -,1844 ,1110 -,4496 -,0128

Ind3 ,0507 ,0371 -,0075 ,1382

Partially standardized indirect effect(s) of X on Y:

Effect BootSE BootLLCI BootULCI

TOTAL -,0822 ,0511 -,1803 ,0205

Ind1 -,0430 ,0428 -,1255 ,0447

Ind2 -,0541 ,0290 -,1186 -,0042

Ind3 ,0149 ,0099 -,0022 ,0374

Indirect effect key:

Ind1 Kontroll -> ctq1ges -> QST07K

Ind2 Kontroll -> m2373 -> QST07K

Ind3 Kontroll -> ctq1ges -> m2373 -> QST07K

*********************** ANALYSIS NOTES AND ERRORS ************************

Level of confidence for all confidence intervals in output:

95,0000

Number of bootstrap samples for percentile bootstrap confidence intervals:

10000

NOTE: A heteroscedasticity consistent standard error and covariance matrix estimator was used.

NOTE: Variables names longer than eight characters can produce incorrect output.

Shorter variable names are recommended.

------ END MATRIX -----

## Tactile control threshold test site

Run MATRIX procedure:

*************** PROCESS Procedure for SPSS Version 3.3 *******************

Written by Andrew F. Hayes, Ph.D. www.afhayes.com

Documentation available in Hayes (2018). www.guilford.com/p/hayes3

**************************************************************************

Model : 6

Y : QST07T

X : Kontroll

M1 : ctq1ges

M2 : m2373

Sample

Size: 228

Custom

Seed: 1

**************************************************************************

OUTCOME VARIABLE:

ctq1ges

Model Summary

R R-sq MSE F(HC3) df1 df2 p

,3186 ,1015 225,2346 24,6753 1,0000 226,0000 ,0000

Model

coeff se(HC3) t p LLCI ULCI

constant 35,8654 1,0184 35,2182 ,0000 33,8587 37,8721

Kontroll 10,0468 2,0225 4,9674 ,0000 6,0613 14,0322

**************************************************************************

OUTCOME VARIABLE:

m2373

Model Summary

R R-sq MSE F(HC3) df1 df2 p

,1665 ,0277 ,0039 2,9940 2,0000 225,0000 ,0521

Model

coeff se(HC3) t p LLCI ULCI

constant ,5561 ,0123 45,3600 ,0000 ,5319 ,5803

Kontroll ,0192 ,0088 2,1879 ,0297 ,0019 ,0365

ctq1ges -,0005 ,0003 -1,7187 ,0870 -,0011 ,0001

**************************************************************************

OUTCOME VARIABLE:

QST07T

Model Summary

R R-sq MSE F(HC3) df1 df2 p

,2689 ,0723 183,7032 4,2974 3,0000 224,0000 ,0057

Model

coeff se(HC3) t p LLCI ULCI

constant 21,9336 10,8200 2,0271 ,0438 ,6116 43,2556

Kontroll 6,3399 2,0585 3,0798 ,0023 2,2833 10,3965

ctq1ges ,0128 ,0746 ,1721 ,8635 -,1342 ,1599

m2373 -35,7752 18,1283 -1,9735 ,0497 -71,4990 -,0515

************************** TOTAL EFFECT MODEL ****************************

OUTCOME VARIABLE:

QST07T

Model Summary

R R-sq MSE F(HC3) df1 df2 p

,2140 ,0458 187,2776 10,2203 1,0000 226,0000 ,0016

Model

coeff se(HC3) t p LLCI ULCI

constant 3,1735 ,2824 11,2372 ,0000 2,6170 3,7300

Kontroll 5,9715 1,8679 3,1969 ,0016 2,2908 9,6521

************** TOTAL, DIRECT, AND INDIRECT EFFECTS OF X ON Y **************

Total effect of X on Y

Effect se(HC3) t p LLCI ULCI c_ps

5,9715 1,8679 3,1969 ,0016 2,2908 9,6521 ,4272

Direct effect of X on Y

Effect se(HC3) t p LLCI ULCI c'_ps

6,3399 2,0585 3,0798 ,0023 2,2833 10,3965 ,4535

Indirect effect(s) of X on Y:

Effect BootSE BootLLCI BootULCI

TOTAL -,3684 ,8754 -2,1038 1,4137

Ind1 ,1290 ,7444 -1,2478 1,7488

Ind2 -,6863 ,4517 -1,7351 -,0192

Ind3 ,1888 ,1463 -,0242 ,5368

Partially standardized indirect effect(s) of X on Y:

Effect BootSE BootLLCI BootULCI

TOTAL -,0264 ,0662 -,1638 ,0912

Ind1 ,0092 ,0533 -,1014 ,1053

Ind2 -,0491 ,0303 -,1184 -,0019

Ind3 ,0135 ,0098 -,0023 ,0362

Indirect effect key:

Ind1 Kontroll -> ctq1ges -> QST07T

Ind2 Kontroll -> m2373 -> QST07T

Ind3 Kontroll -> ctq1ges -> m2373 -> QST07T

*********************** ANALYSIS NOTES AND ERRORS ************************

Level of confidence for all confidence intervals in output:

95,0000

Number of bootstrap samples for percentile bootstrap confidence intervals:

10000

NOTE: A heteroscedasticity consistent standard error and covariance matrix estimator was used.

NOTE: Variables names longer than eight characters can produce incorrect output.

Shorter variable names are recommended.

------ END MATRIX -----

## Pressure pain threshold control site

Run MATRIX procedure:

*************** PROCESS Procedure for SPSS Version 3.3 *******************

Written by Andrew F. Hayes, Ph.D. www.afhayes.com

Documentation available in Hayes (2018). www.guilford.com/p/hayes3

**************************************************************************

Model : 6

Y : QST13K

X : Kontroll

M1 : ctq1ges

M2 : m2373

Sample

Size: 227

Custom

Seed: 1

**************************************************************************

OUTCOME VARIABLE:

ctq1ges

Model Summary

R R-sq MSE F(HC3) df1 df2 p

,3164 ,1001 226,0244 24,2992 1,0000 225,0000 ,0000

Model

coeff se(HC3) t p LLCI ULCI

constant 35,9246 1,0255 35,0320 ,0000 33,9038 37,9453

Kontroll 9,9876 2,0261 4,9294 ,0000 5,9950 13,9802

**************************************************************************

OUTCOME VARIABLE:

m2373

Model Summary

R R-sq MSE F(HC3) df1 df2 p

,1688 ,0285 ,0039 3,0741 2,0000 224,0000 ,0482

Model

coeff se(HC3) t p LLCI ULCI

constant ,5550 ,0123 45,2389 ,0000 ,5309 ,5792

Kontroll ,0198 ,0088 2,2518 ,0253 ,0025 ,0370

ctq1ges -,0005 ,0003 -1,6827 ,0938 -,0011 ,0001

**************************************************************************

OUTCOME VARIABLE:

QST13K

Model Summary

R R-sq MSE F(HC3) df1 df2 p

,5037 ,2538 10211,0441 24,5128 3,0000 223,0000 ,0000

Model

coeff se(HC3) t p LLCI ULCI

constant 391,9931 76,0602 5,1537 ,0000 242,1043 541,8820

Kontroll -113,7139 14,4548 -7,8669 ,0000 -142,1993 -85,2285

ctq1ges -,2085 ,5138 -,4057 ,6853 -1,2210 ,8040

m2373 -50,8270 126,4047 -,4021 ,6880 -299,9277 198,2736

************************** TOTAL EFFECT MODEL ****************************

OUTCOME VARIABLE:

QST13K

Model Summary

R R-sq MSE F(HC3) df1 df2 p

,5025 ,2525 10137,8058 74,4437 1,0000 225,0000 ,0000

Model

coeff se(HC3) t p LLCI ULCI

constant 357,2328 8,0832 44,1942 ,0000 341,3042 373,1613

Kontroll -116,5391 13,5070 -8,6281 ,0000 -143,1554 -89,9227

************** TOTAL, DIRECT, AND INDIRECT EFFECTS OF X ON Y **************

Total effect of X on Y

Effect se(HC3) t p LLCI ULCI c_ps

-116,5391 13,5070 -8,6281 ,0000 -143,1554 -89,9227 -1,0029

Direct effect of X on Y

Effect se(HC3) t p LLCI ULCI c'_ps

-113,7139 14,4548 -7,8669 ,0000 -142,1993 -85,2285 -,9786

Indirect effect(s) of X on Y:

Effect BootSE BootLLCI BootULCI

TOTAL -2,8251 5,7819 -13,9830 8,8592

Ind1 -2,0821 5,0304 -11,9470 7,9075

Ind2 -1,0042 2,6861 -6,8240 4,2943

Ind3 ,2611 ,7573 -1,1235 2,0562

Partially standardized indirect effect(s) of X on Y:

Effect BootSE BootLLCI BootULCI

TOTAL -,0243 ,0497 -,1191 ,0771

Ind1 -,0179 ,0433 -,1027 ,0673

Ind2 -,0086 ,0231 -,0582 ,0374

Ind3 ,0022 ,0065 -,0098 ,0175

Indirect effect key:

Ind1 Kontroll -> ctq1ges -> QST13K

Ind2 Kontroll -> m2373 -> QST13K

Ind3 Kontroll -> ctq1ges -> m2373 -> QST13K

*********************** ANALYSIS NOTES AND ERRORS ************************

Level of confidence for all confidence intervals in output:

95,0000

Number of bootstrap samples for percentile bootstrap confidence intervals:

10000

NOTE: A heteroscedasticity consistent standard error and covariance matrix estimator was used.

NOTE: Variables names longer than eight characters can produce incorrect output.

Shorter variable names are recommended.

------ END MATRIX -----

## Pressure pain threshold test site

Run MATRIX procedure:

*************** PROCESS Procedure for SPSS Version 3.3 *******************

Written by Andrew F. Hayes, Ph.D. www.afhayes.com

Documentation available in Hayes (2018). www.guilford.com/p/hayes3

**************************************************************************

Model : 6

Y : QST13T

X : Kontroll

M1 : ctq1ges

M2 : m2373

Sample

Size: 227

Custom

Seed: 1

**************************************************************************

OUTCOME VARIABLE:

ctq1ges

Model Summary

R R-sq MSE F(HC3) df1 df2 p

,3164 ,1001 226,0244 24,2992 1,0000 225,0000 ,0000

Model

coeff se(HC3) t p LLCI ULCI

constant 35,9246 1,0255 35,0320 ,0000 33,9038 37,9453

Kontroll 9,9876 2,0261 4,9294 ,0000 5,9950 13,9802

**************************************************************************

OUTCOME VARIABLE:

m2373

Model Summary

R R-sq MSE F(HC3) df1 df2 p

,1688 ,0285 ,0039 3,0741 2,0000 224,0000 ,0482

Model

coeff se(HC3) t p LLCI ULCI

constant ,5550 ,0123 45,2389 ,0000 ,5309 ,5792

Kontroll ,0198 ,0088 2,2518 ,0253 ,0025 ,0370

ctq1ges -,0005 ,0003 -1,6827 ,0938 -,0011 ,0001

**************************************************************************

OUTCOME VARIABLE:

QST13T

Model Summary

R R-sq MSE F(HC3) df1 df2 p

,5293 ,2802 16337,2066 29,7496 3,0000 223,0000 ,0000

Model

coeff se(HC3) t p LLCI ULCI

constant 242,3256 79,0115 3,0670 ,0024 86,6208 398,0303

Kontroll -161,9781 17,6217 -9,1920 ,0000 -196,7044 -127,2518

ctq1ges ,2926 ,6682 ,4379 ,6619 -1,0242 1,6093

m2373 180,7526 150,8983 1,1978 ,2322 -116,6166 478,1218

************************** TOTAL EFFECT MODEL ****************************

OUTCOME VARIABLE:

QST13T

Model Summary

R R-sq MSE F(HC3) df1 df2 p

,5236 ,2742 16327,6265 83,3503 1,0000 225,0000 ,0000

Model

coeff se(HC3) t p LLCI ULCI

constant 349,8190 10,4176 33,5796 ,0000 329,2904 370,3475

Kontroll -156,4136 17,1325 -9,1296 ,0000 -190,1743 -122,6529

************** TOTAL, DIRECT, AND INDIRECT EFFECTS OF X ON Y **************

Total effect of X on Y

Effect se(HC3) t p LLCI ULCI c_ps

-156,4136 17,1325 -9,1296 ,0000 -190,1743 -122,6529 -1,0452

Direct effect of X on Y

Effect se(HC3) t p LLCI ULCI c'_ps

-161,9781 17,6217 -9,1920 ,0000 -196,7044 -127,2518 -1,0824

Indirect effect(s) of X on Y:

Effect BootSE BootLLCI BootULCI

TOTAL 5,5645 6,9139 -7,0402 20,5741

Ind1 2,9220 6,6745 -8,9850 17,6022

Ind2 3,5712 3,7330 -2,3313 12,3405

Ind3 -,9287 1,1180 -3,6560 ,7226

Partially standardized indirect effect(s) of X on Y:

Effect BootSE BootLLCI BootULCI

TOTAL ,0372 ,0465 -,0467 ,1384

Ind1 ,0195 ,0444 -,0617 ,1154

Ind2 ,0239 ,0256 -,0150 ,0853

Ind3 -,0062 ,0075 -,0247 ,0045

Indirect effect key:

Ind1 Kontroll -> ctq1ges -> QST13T

Ind2 Kontroll -> m2373 -> QST13T

Ind3 Kontroll -> ctq1ges -> m2373 -> QST13T

*********************** ANALYSIS NOTES AND ERRORS ************************

Level of confidence for all confidence intervals in output:

95,0000

Number of bootstrap samples for percentile bootstrap confidence intervals:

10000

NOTE: A heteroscedasticity consistent standard error and covariance matrix estimator was used.

NOTE: Variables names longer than eight characters can produce incorrect output.

Shorter variable names are recommended.

------ END MATRIX -----
